# Transient absorption spectroscopy of the electron transfer step in the photochemically activated polymerizations of *N*-ethylcarbazole and 9-phenylcarbazole†

**DOI:** 10.1039/d1cp03137f

**Published:** 2021-08-05

**Authors:** Georgia L. Thornton, Ryan Phelps, Andrew J. Orr-Ewing

**Affiliations:** School of Chemistry, University of Bristol, Cantock's Close Bristol BS8 1TS UK a.orr-ewing@bristol.ac.uk

## Abstract

The polymerization of photoexcited N-ethylcarbazole (N-EC) in the presence of an electron acceptor begins with an electron transfer (ET) step to generate a radical cation of N-EC (N-EC˙^+^). Here, the production of N-EC˙^+^ is studied on picosecond to nanosecond timescales after N-EC photoexcitation at a wavelength *λ*_ex_ = 345 nm using transient electronic and vibrational absorption spectroscopy. The kinetics and mechanisms of ET to diphenyliodonium hexafluorophosphate (Ph_2_I^+^PF_6_^−^) or para-alkylated variants are examined in dichloromethane (DCM) and acetonitrile (ACN) solutions. The generation of N-EC˙^+^ is well described by a diffusional kinetic model based on Smoluchowski theory: with Ph_2_I^+^PF_6_^−^, the derived bimolecular rate coefficient for ET is *k*_ET_ = (1.8 ± 0.5) × 10^10^ M^−1^ s^−1^ in DCM, which is consistent with diffusion-limited kinetics. This ET occurs from the first excited singlet (S_1_) state of N-EC, in competition with intersystem crossing to populate the triplet (T_1_) state, from which ET may also arise. A faster component of the ET reaction suggests pre-formation of a ground-state complex between N-EC and the electron acceptor. In ACN, the contribution from pre-reaction complexes is smaller, and the derived ET rate coefficient is *k*_ET_ = (1.0 ± 0.3) × 10^10^ M^−1^ s^−1^. Corresponding measurements for solutions of photoexcited 9-phenylcarbazole (9-PC) and Ph_2_I^+^PF_6_^−^ give *k*_ET_ = (5 ± 1) × 10^9^ M^−1^ s^−1^ in DCM. Structural modifications of the electron acceptor to increase its steric bulk reduce the magnitude of *k*_ET_: methyl and *t*-butyl additions to the para positions of the phenyl rings (para Me_2_Ph_2_I^+^PF_6_^−^ and *t*-butyl-Ph_2_I^+^PF_6_^−^) respectively give *k*_ET_ = (1.2 ± 0.3) × 10^10^ M^−1^ s^−1^ and *k*_ET_ = (5.4 ± 1.5) × 10^9^ M^−1^ s^−1^ for reaction with photoexcited N-EC in DCM. These reductions in *k*_ET_ are attributed to slower rates of diffusion or to steric constraints in the ET reaction.

## Abstract 2

Transient absorption spectroscopy of electron transfer reactions between a carbazole and an iodonium salt reveals structure and solvent-dependent kinetic and mechanistic details important to initiation of polymerization.

## Graphical abstract

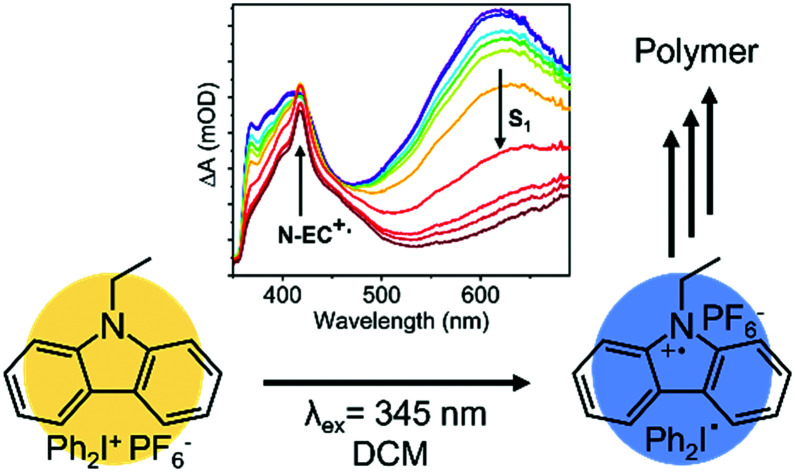

## 1. Introduction

**Fig. 1:**
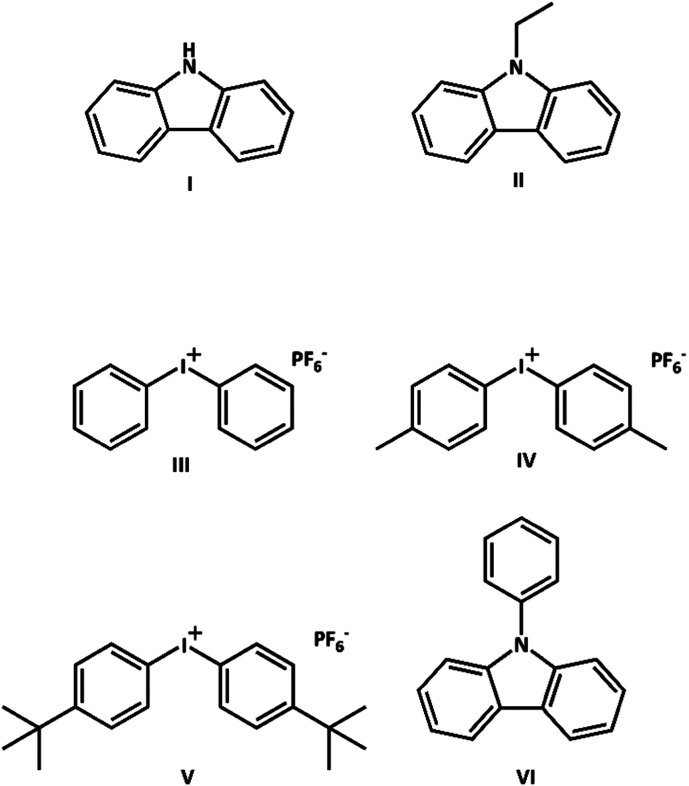
Structures of (I) carbazole, (II) *N*-ethylcarbazole (N-EC), (III) Ph_2_I^+^PF_6_^−^, (IV) Me_2_Ph_2_I^+^PF_6_^−^, (V) *t*-butyl-Ph_2_I^+^PF_6_^−^ and (VI) 9-phenylcarbazole (9-PC).

Photoinduced electron transfer (ET) reactions are finding widespread application in modern synthesis of organic molecules and polymers, with ultraviolet (UV) or visible (Vis) light absorption by a reagent or a photoredox catalyst offering a convenient method to activate the reaction mixture.^1–10^ Carbazoles, the simplest of which is shown in Fig. 1(I), are a class of molecules that readily undergo ET from their excited electronic states.^1,2^ They are known to be photoinitiators in cationic and radical polymerization of monomers, depending on the species present in solution.^11–13^ The conjugated nature of a carbazole-based polymer allows electrical charge to migrate along its backbone, and this conducting characteristic has proven important in the development of batteries, solar cells and other electronic components.^1,2,14–16^ Compounds possessing a carbazole moiety are attractive for technological applications because they can withstand high temperatures,^2,14,16,17^ and are readily soluble when modified with alkyl chains.^1,12,18^

Yagci and co-workers have explored the use of carbazole derivatives as photoinitiators in radical and cationic polymerization.^11–13^ Structural modification of the carbazoles shifted their absorptions to the visible region,^11,12^ with potential benefits for sustainable chemistry if photoinitiation can be driven by sunlight in place of energy-intensive artificial light sources.^11,19^ Sari *et al.* recently reported the use of *N*-ethylcarbazole (N-EC), as shown in Fig. 1(II), in a photoredox polymerization scheme.^1^ Despite being structurally simpler than other studied carbazoles,^11–13^ N-EC acts as the monomer in a polymerization reaction in the presence of an electron acceptor (EA), without the need for a metal complex as a catalyst. This behaviour reduces the complexity of a reaction mixture by minimizing the number of participating reagents. The reaction simplification decreases the costs of product purification, and eliminates the use of metal reagents which are commonly found in polymerization catalysts, but can be difficult to remove and may be toxic if left in the polymer product.^4,6,7,20^ The other reagent required is diphenyliodonium hexafluorophosphate (Ph_2_I^+^PF_6_^−^, Fig. 1(III)), which was previously shown to induce cation formation in a radical carbazole species.^12,21^ The Ph_2_I^+^ moiety acts as an EA, with single ET from photoexcited N-EC generating a radical cation (N-EC˙^+^). The PF_6_^−^ is a spectator ion, chosen for its low nucleophilicity to avoid reaction with N-EC˙^+^.^1,21^ The formation of N-EC˙^+^ in this way might initiate polymerization *via* either radical or cationic polymerization mechanisms. When N-EC is irradiated with near-UV light in the presence of Ph_2_I^+^PF_6_^−^, the polymerization proceeds *via* a step-growth mechanism.^1^ Step-growth polymerization generates polymers with a high poly-dispersity index (PDI), which can be useful in some commercial applications because the distribution of molecular weights introduces species with different properties into the same material.^22^

**Scheme 1:**
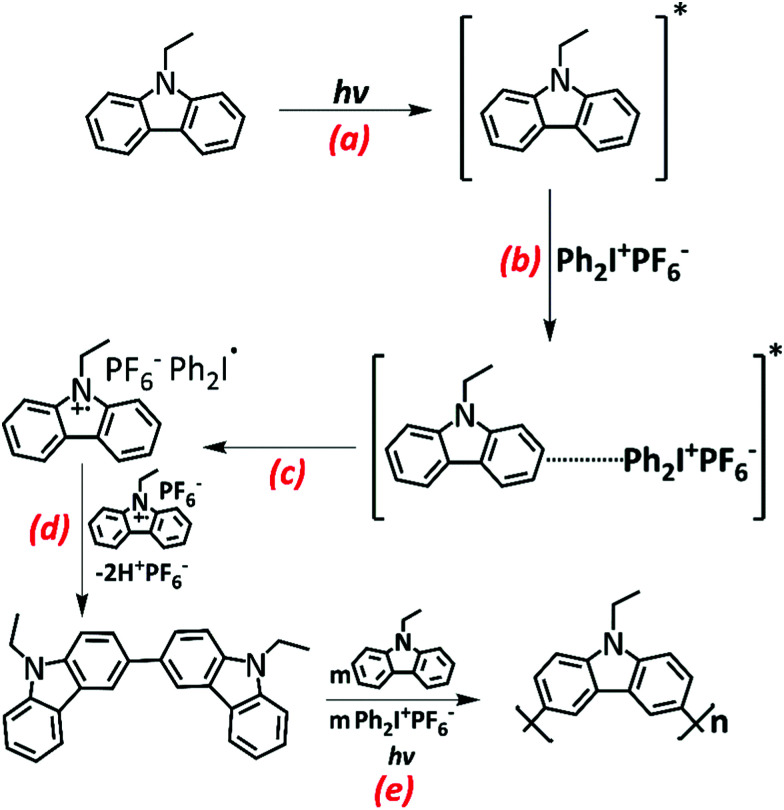
Proposed mechanism for the step-growth polymerization of N-EC in the presence of an EA, Ph_2_I^+^PF_6_^−^.^1^ The mechanism involves five key steps: N-EC photo-excitation (a), followed by complexation with GS Ph_2_I^+^PF_6_^−^ (b), allowing intermolecular ET to form a radical cation N-EC˙^+^ (c) Dimerization of two radical cationic species forms a neutral dimeric compound (d) by elimination of 2H^+^. Repetition of these steps forms a polymer (e).

Sari *et al.* proposed the mechanism shown in Scheme 1 for the photo-induced step-growth polymerization of N-EC in the presence of Ph_2_I^+^PF_6_^−^. Near-UV excitation of N-EC (a) is followed by exciplex formation with ground-state (GS) Ph_2_I^+^PF_6_^−^ (b), followed by ET to form N-EC˙^+^ (c). The subsequent dimerization of two N-EC˙^+^ radicals releases two H^+^ ions favourably at carbon sites 3 and 6 (d).^2,18,23^ This deprotonation occurs because of radical delocalization into the benzene ring.^2^ Continued propagation of the chain forms a polymer (e).^1^ UV-Visible spectroscopy of solutions of the starting materials suggested that N-EC must be in an electronically excited state to interact with Ph_2_I^+^PF_6_^−^ because there was no observed spectroscopic evidence for a GS complex.^1^

Only a few prior studies have investigated the photoexcitation of N-EC using time-resolved spectroscopy, and the detailed kinetics and mechanism of polymerization remain largely unexplored. Transient absorption spectroscopy studies of N-EC excited at *λ*_ex_ = 308 nm, with nanosecond time resolution, identified two excited state absorption (ESA) bands centred at 628 nm and 392 nm.^24^ These bands were assigned to absorption from the S_1_ and T_1_ states of N-EC, respectively. S_1_ state assignment was supported by comparisons with similar studies of carbazole,^25^ whereas T_1_ assignment was confirmed by oxygen quenching.^24^

The objectives of the current study are to contrast the ultrafast photochemistry of excited state N-EC in the presence and absence of Ph_2_I^+^PF_6_^−^, and to explore the effects of replacing the *N*-ethyl substituent by an *N*-phenyl group using 9-phenylcarbazole (9-PC) (see Fig. 1(VI)). Karon *et al.* previously concluded that because the *N*-phenyl group is twisted with respect to the carbazole body, and therefore not conjugated to the remainder of the molecule, dimerization will still occur at positions 3 and 6.^23^ However, the presence of the *N*-phenyl ring will modify the electronic energies and characters of the carbazole excited states involved in ET reactions, and hence the ET rates.

Transient electronic absorption spectroscopy (TEAS) and transient vibrational absorption spectroscopy (TVAS) are used to observe the initial steps in the mechanism of polymerization of N-EC following 345 nm sample excitation, corresponding to photoabsorption to the S_1_ state of N-EC, intersystem crossing (ISC) to populate the T_1_ state, and excited-state intermolecular ET reaction with an EA. These measurements test the reaction scheme proposed by Sari *et al.*^1^ and quantify the reaction kinetics while distinguishing whether ET occurs from the S_1_ or T_1_ state. To examine steric effects on the ET rates, the experimental studies reported here use two EAs in addition to Ph_2_I^+^PF_6_^−^, namely bis(4-methylphenyl)iodonium hexafluorophosphate (Me_2_Ph_2_I^+^PF_6_^−^) and bis(4-*tert*-butylphenyl)iodonium hexafluorophosphate (*t*-butyl-Ph_2_I^+^PF_6_^−^) (see Fig. 1(IV) and (V)).^26,27^ Experiments conducted in dichloromethane (DCM) and acetonitrile (ACN) explore solvent effects on the initial steps in the polymerization of N-EC with Ph_2_I^+^PF_6_^−^.

## 2. Methodology

### 2.1 Experimental measurements

The dynamics of photoexcited N-EC and 9-PC, and their ET reactions with Ph_2_I^+^ and other EAs were probed using ultrafast transient absorption spectroscopy. Characteristic peaks in the transient electronic and vibrational absorption spectra were used to reveal the population dynamics of S_1_ and T_1_ states of the carbazoles, and the growth of products from intermolecular ET.

Details of the steady-state spectroscopy methods used to characterize the UV-visible and infrared (IR) absorption bands of the starting solutions can be found in Section S1 of the ESI.† Full descriptions of the TEAS and TVAS methods applied are provided elsewhere,^28–30^ with a summary given here. Solutions were made to the desired concentrations of the carbazole and other reagents, and a peristaltic pump continuously circulated 10 ml samples through a Harrick cell with calcium fluoride (CaF_2_) windows separated by 250 μm Teflon spacers. The cell was rastered horizontally and vertically to avoid sample photodamage or polymer deposition on the windows. All measurements were made at a laboratory temperature of 20 °C.

The 800 nm output of an amplified Titanium-sapphire laser system (Coherent Vitara-S and Legend Elite HE+; 5 W, 1 kHz, 35 fs duration pulses) was split into three beams, two of which (≤2.45W each) pumped optical parametric amplifiers (OPAs), with the remaining 0.1 W in the third beam used to generate a white light continuum (WLC) spanning ∼350–700 nm by focusing into a CaF_2_ window. This WLC was used as a probe for the TEAS measurements reported here. The first OPA produced near-UV pulses of wavelength 345 nm for the sample excitation. Difference frequency generation using the signal and idler outputs of the second OPA provided IR probe pulses spanning the wavenumber range 1435–1635 cm^−1^ for TVAS. The UV pump laser energy was regulated using a combination of a half-wave plate and polarizer and set to be ∼500 nJ per pulse for TEAS and ∼600 nJ for TVAS experiments.

Delays between the UV pump and WLC or IR probe pulses of up to 1.3 ns were controlled by a delay stage in the optical line for the pump laser pulses. A mechanical chopper blocked every second pump pulse to generate sequential pump-on and pump-off measurements for difference-spectrum analysis. The UV light was passed through a polarizer set to the magic angle of 54.7° with respect to the probe laser polarization to eliminate the effects of rotational diffusion on transient spectra, before being focused into the sample using a *f* = 200 mm CaF_2_ focal-length lens. The mid-IR or WLC probe was focused and overlapped with the larger-radius pump beam at the centre of the Harrick cell using a *f* = 75 mm concave aluminium mirror. The transmitted probe light was collected and dispersed in a spectrograph. TEAS experiments used an Andor spectrometer (Shamrock 163) fitted with a 1024-element photodiode array (Entwicklungsbüro Stresing), whereas TVAS measurements used a HORIBA Scientific spectrometer (iHR320) with a 128-element MCT array (Infrared Associates Inc., MCT-10-128). A small portion of the IR probe was split from the main beam prior to passing through the sample and was directed into a matching spectrometer to provide a reference for shot-to-shot noise reduction. Prior to the sample, the TEAS probe beam passed through a 2 mm cuvette containing a concentrated solution of copper sulfate (≥99.9%, VWR Chemicals) in deionized water to absorb any residual 800 nm light from WLC generation. A further 2 mm cuvette containing a >7 mM solution of N-EC in DCM was placed between the sample and the Andor spectrometer to minimize the effects of pump laser scatter on TEAS measurements. The instrument response function (IRF) for TEAS measurements was ∼110–120 fs, as determined previously by Roberts *et al.*^28^

DCM (≥99.9%, Merck, Uvasol®) and ACN (≥99.9%, Merck, Uvasol®) were used without additional purification to prepare 7 mM solutions of N-EC (99%, Alfa Aesar) or 9-PC (99%, Alfa Aesar). In measurements involving the EA, a 7 mM solution of N-EC or 9-PC was prepared containing 7 mM, 14 mM, 28 mM, 42 mM, 56 mM or 84 mM Ph_2_I^+^PF_6_^−^ (98%, Alfa Aesar), Me_2_Ph_2_I^+^PF_6_^−^ (98%, Sigma-Aldrich), or *t*-butyl-Ph_2_I^+^PF_6_^−^ (98%, Sigma-Aldrich). For the synthesis of poly(*N*-EC) Sari *et al.* used a 1 : 2 ratio of N-EC : Ph_2_I^+^PF_6_^−^ (200 mM: 400 mM) for the polymerization method, with our concentrations adjusted to yield an optical density of approximately 0.7–0.8.^1^ HPLC grade (≥99.9%) ACN and lab grade (≥99.9%) DCM were used for steady state spectroscopy measurements. Samples were not degassed or purged of dissolved oxygen because, on the ≤1.3 ns maximum timescales of the measurements, radical reactions with O_2_ or excited state quenching are negligible. In control experiments, TEAS and TVAS spectra were recorded for 345 nm excitation of 14 mM and 84 mM solutions of Ph_2_I^+^PF_6_^−^ in DCM, and 14 mM solutions of Me_2_Ph_2_I^+^PF_6_^−^ and *t*-butyl-Ph_2_I^+^PF_6_^−^ in DCM, but no transient absorption features were observed in our UV-Vis and IR spectral windows.

All recorded transient absorption spectra were analysed using the KOALA software package^31^ to decompose the spectra into their component absorption bands and extract time-dependent band intensities, which were fitted to kinetic models.^32–34^ Further details of the methods of analysis can be found below and in the ESI† (Section S5).

### 2.2 Computation of structures and spectra

Experimental spectra were assigned with the aid of quantum chemistry calculations performed using the Gaussian 09W software package.^35^ In summary, density functional theory (DFT) calculations for N-EC and 9-PC were performed using the B3LYP functional with a 6-31+G(d) basis set without correction for solvent effects. GS geometry optimizations and frequency calculations were performed to predict the steady-state IR spectra of N-EC and 9-PC. The computational method was validated by comparing these unscaled frequency calculations with observed band positions in FTIR spectra of N-EC solutions. Correction factors for computed harmonic vibrational frequencies lying within the TVAS probe wavenumber window were derived from these comparisons. A scaling of 0.973 was established for N-EC using the measured vibrational band at 1454 cm^−1^ and the corresponding computational value of 1494 cm^−1^, and this scaling factor was then applied to the computed vibrational frequencies for excited state species and reaction products.

The optimized GS geometries were used in time-dependent (TD) DFT calculations of electronic transition oscillator strengths and vertical excitation energies for both N-EC and 9-PC, to assist with assignment of steady-state and transient spectral features. All excited-state calculations were performed for isolated, gas phase molecules without correction for solvent effects. Further excited-state optimization calculations were performed for N-EC and 9-PC to determine the energies of their S_1_ states after structural relaxation from the Franck–Condon region.

Quantum chemistry calculations were also used to characterize the ground-state structures of pairwise complexes involving the chosen carbazoles and electron acceptors in different solvents, and to predict the wavelengths of their charge-transfer excitation bands. Both DFT and TDDFT calculations were performed using a B3LYP functional with a 6-31+G(d) basis set for carbon, hydrogen and nitrogen atoms and a LANL2DZ basis set for the iodine atoms in the EA. An IEFPCM solvent model was used to represent the solvent environment. The B3LYP functional was sufficient for ground-state optimizations,^36^ but interaction energies may be underestimated.^37^ Although the B3LYP functional has been used to model CT interactions successfully between two organic molecules,^38^ addition of dispersion corrections might be necessary.^39,40^ However, the current treatment of the CT interaction between an aromatic system and a cation without such dispersion corrections was adequate for our purposes.^41^

## 3. Results and discussion

### 3.1. Steady-state spectroscopy

**Fig. 2:**
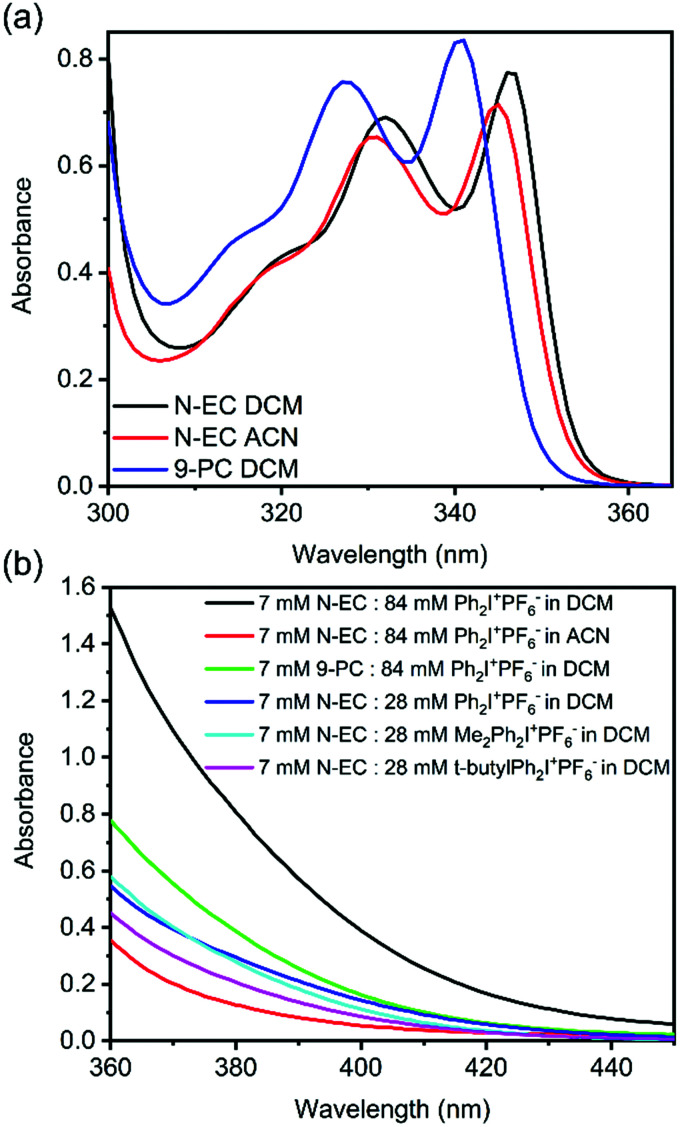
Steady-state UV-Vis absorption spectra of: (a) 7 mM solutions of N-EC in DCM (black) and ACN (red), and 9-PC in DCM (blue); (b) absorption bands for the GS complexes between a carbazole (either N-EC or 9-PC) and an EA (Ph_2_I^+^PF_6_^−^, Me_2_Ph_2_I^+^PF_6_^−^, or *t*-butyl-Ph_2_I^+^PF_6_^−^) at wavelengths extending beyond the uncomplexed carbazole absorption bands. Spectra of single-component solutions of the corresponding carbazole and EA in the same solvent have been subtracted to isolate the spectra of the complexes. A sample pathlength of 250 μm used for the spectra in panel (a) was increased to 1 cm for the spectra in (b) to enhance the weak, long-wavelength absorption features.

Steady state UV-Vis absorption spectra of N-EC in DCM and ACN solutions, and of 9-PC in DCM, are shown in Fig. 2. Guided by our quantum chemistry calculations (ESI,† Section S6, Fig. S18 and S19), the absorption band centred at 346 nm for N-EC in DCM is assigned to the origin band of the S_1_ ← S_0_ (π* ← π) (oscillator strength, *f* = 0.037) electronic excitation, and appears to show vibronic structure with a further peak at 332 nm.^42^ Below 300 nm, the onset of absorption to the S_2_ state is observed (*f* = 0.096).^42^ An excitation wavelength of 345 nm was chosen for all transient absorption spectroscopy studies of solutions of N-EC in DCM and ACN, and for 9-PC in DCM because the changes in solvent and choice of carbazole compound did not significantly alter the locations of the absorption maxima as seen in Fig. 2 and the ESI,† Section S2 and Fig. S1–S4.

Although Yagci and co-workers suggested that there was no evidence of GS complexation between N-EC and Ph_2_I^+^PF_6_^−^ in DCM, we observed that colourless solutions of 7 mM N-EC in DCM and 84 mM Ph_2_I^+^PF_6_^−^ in DCM became yellow when combined. This observation signifies that solute–solute interactions extend the electronic absorption bands into the visible region, as supported by the appearance of a band shown in Fig. 2(b) extending from wavelengths below 360 nm to beyond 400 nm in the UV-Vis spectrum of this mixture. We assign this new band to charge transfer (CT) in a GS complex^43^ between N-EC and Ph_2_I^+^PF_6_^−^, with this assignment supported by a computed gas-phase CT band for N-EC and Ph_2_I^+^PF_6_^−^ at 324 nm (ESI,† Section S4 and Fig. S11). Computational limitations prevented the corresponding calculations in DCM; however, the gas-phase calculations should provide a reasonable approximation for the spectrum in this low polarity solvent. A similar band was observed for N-EC and Ph_2_I^+^PF_6_^−^ mixtures in ACN (Fig. 2(b)), but the smaller absorbance suggests a lower propensity for GS complexation in this solvent. Computational analysis of this system predicts a CT band at 321 nm (ESI,† Section S4 and Fig. S12). Spectroscopic analysis of a solution of 7 mM 9-PC and 84 mM Ph_2_I^+^PF_6_^−^ in DCM (Fig. 2(b)) also points to a degree of GS complexation. Similar evidence for GS complexation to N-EC was obtained when Ph_2_I^+^PF_6_^−^ was replaced with Me_2_Ph_2_I^+^PF_6_^−^ or *t*-butyl-Ph_2_I^+^PF_6_^−^ in DCM solutions, with calculations for the latter complex indicating a CT band at 322 nm (ESI,† Section S4 and Fig. S13).

The computed oscillator strengths of the CT bands were used to relate the strengths of the experimentally observed absorption features in Fig. 2(b) to the relative propensities for complex formation in the different solutions. As is seen in Table S1 of the ESI,† the combined experimental and computational data point to a higher concentration of N-EC–Ph_2_I^+^PF_6_^−^ complexes in DCM than in ACN, and the addition of *t*-butyl groups to the EA also reduces complexation. Unfortunately, measurement of transient absorption spectra following selective excitation of the ground-state complexes at wavelengths longer than 360 nm was not possible because of the weakness of their absorption bands.

FTIR spectroscopy of a 7 mM N-EC and 14 mM Ph_2_I^+^PF_6_^−^ solution in DCM revealed strong IR signatures of Ph_2_I^+^PF_6_^−^ at 1582 cm^−1^, 1562 cm^−1^, 1469 cm^−1^, and 1445 cm^−1^, and of N-EC at 1628 cm^−1^, 1600 cm^−1^, 1485 cm^−1^, 1471 cm^−1^, 1458 cm^−1^, and 1454 cm^−1^ (ESI,† Section S2 and Fig. S5). These spectral bands are used to assign GS bleach (GSB) features observed in TVAS measurements in the range 1435–1635 cm^−1^. The strong absorption bands of ACN in this region prevented TVAS measurements in this solvent.

Oxidation of N-EC with ferric chloride (FeCl_3_) generated N-EC˙^+^ and steady-state UV-Vis spectroscopy identified a narrow band at 395 nm and a broader absorption from 600–800 nm that we assign to this radical cation. Example spectra are shown in the ESI,† Section S6 and Fig. S18. These spectroscopic signatures of N-EC˙^+^ were used to track its formation in ET reactions monitored by TEAS spanning the near-UV and visible regions. Similar features were also observed for 9-PC radical cation (9-PC˙^+^) formation (see ESI,† Section S6 and Fig. S19). Computed electronic absorption frequencies compared well with the observed N-EC˙^+^ and 9-PC˙^+^ bands. Assignments for the TEAS data discussed below are based on these observations from steady-state UV-Vis spectroscopy, together with DFT calculations (see Section S6 and Fig. S18, S19 of ESI†), and the kinetic behaviour of the spectral features. Absorption signatures in TVAS were compared with computed IR absorption frequencies for N-EC˙^+^ (see Section S6 and Fig. S17 in ESI†). These comparisons, and the observed time-dependent changes in band intensities, guided our TVAS band assignments.

### 3.2. Transient absorption spectroscopy of N-EC and 9-PC in DCM

**Fig. 3:**
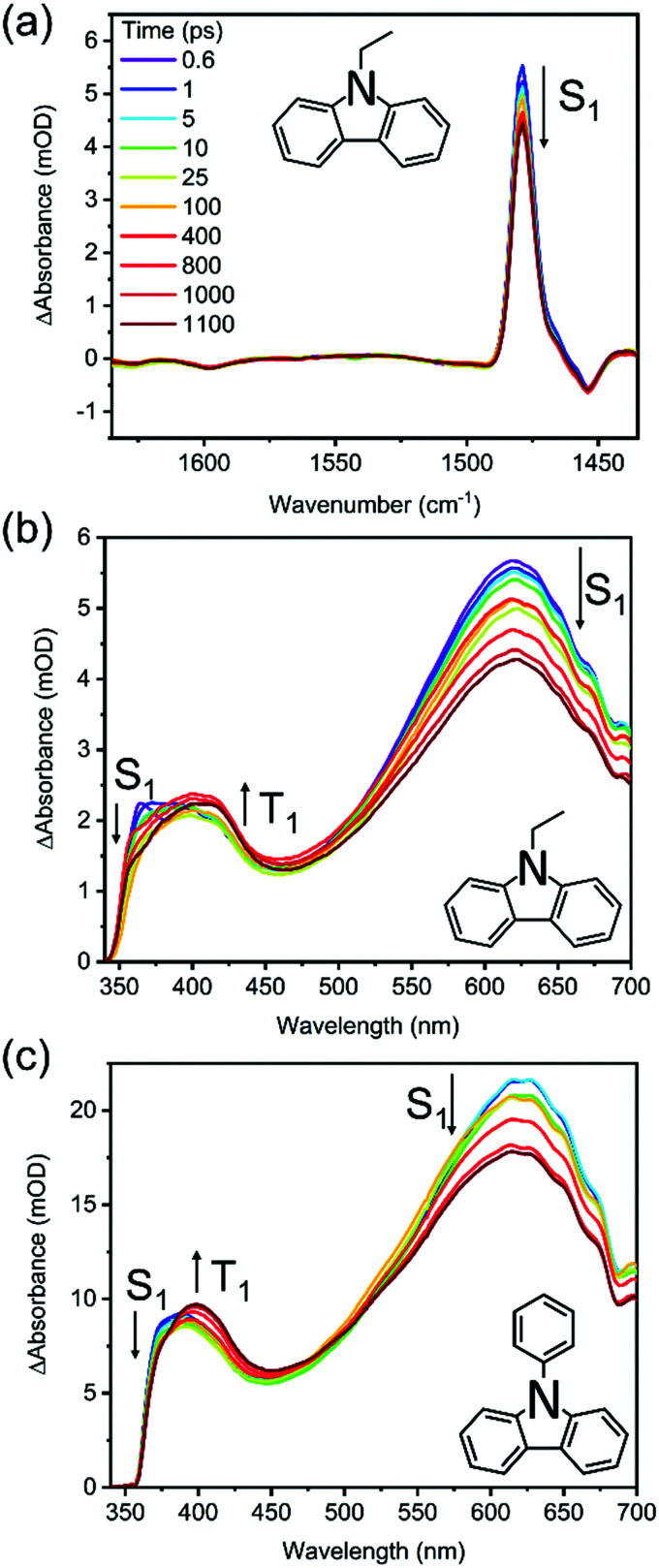
Transient absorption spectra obtained at selected time delays following 345 nm photoexcitation of a 7 mM solution of N-EC or 9-PC in DCM: (a) TVAS of N-EC; (b) TEAS of N-EC; (c) TEAS of 9-PC. The inset colour key identifies the time delays and black arrows show directions of change of transient features.

TVAS and TEAS spectra obtained following UV excitation at 345 nm of a solution of 7 mM N-EC in DCM are shown in Fig. 3(a) and (b). TEAS measurements were also made for 7 mM solutions of N-EC in ACN and 9-PC in DCM. In the TEAS spectra, features arising at wavelengths below 350 nm are not observed because of the fall-off in the intensity of the WLC probe. The methods used for spectral decomposition to extract time-dependent intensities of contributing absorption features are illustrated in the ESI,† Section S5 (Fig. S14–S16).

**Fig. 4:**
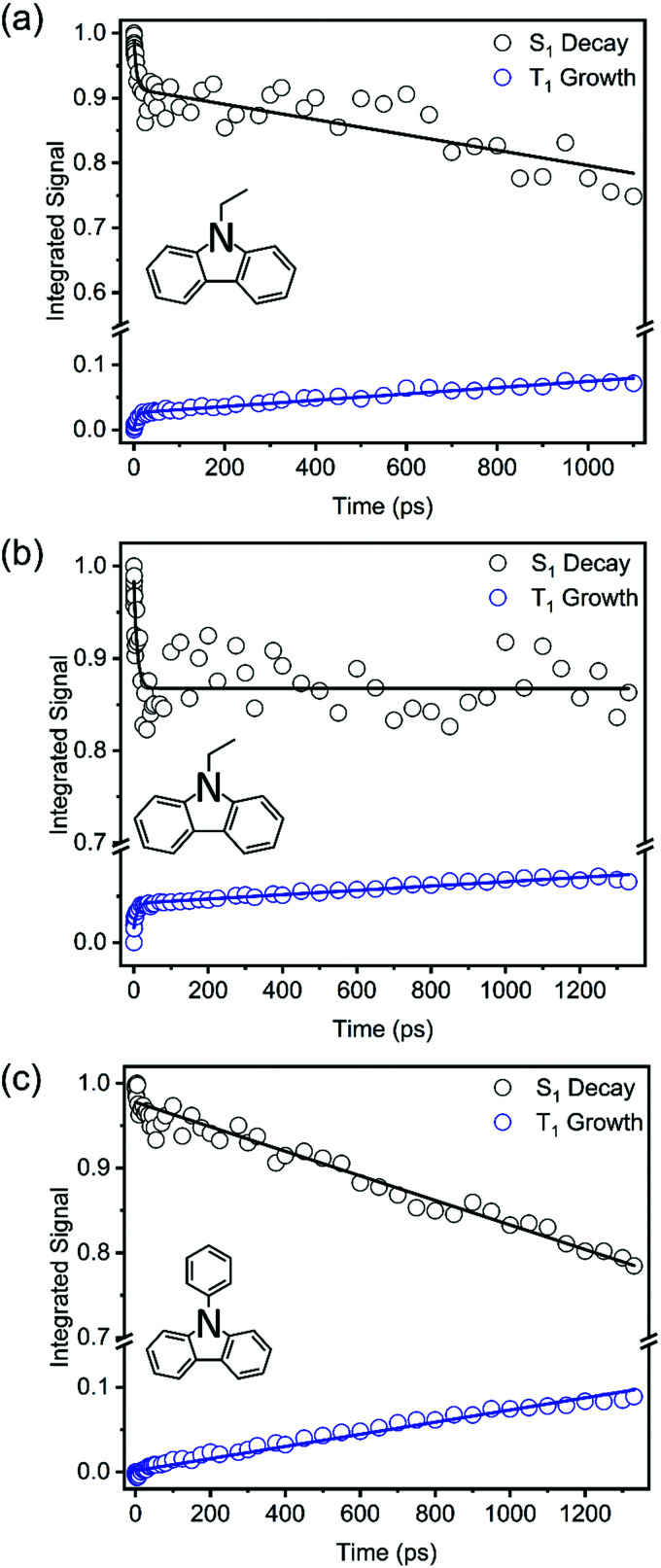
Kinetic plots obtained from TEA spectra for 345 nm photoexcitation of: (a) 7 mM N-EC in DCM; (b) 7 mM N-EC in ACN; and (c) 7 mM 9-PC in DCM. Coloured symbols represent S_1_ ESA decay (black) and T_1_ ESA growth (blue). Solid lines are global fits to all the data within a panel to extract the time constants discussed in the main text.

In prior work by Hiyoshi *et al.*,^24^ and confirmed here, TEAS of N-EC produced time-evolving spectra with two significant features in the near-UV to visible region. A broad, decaying feature centred near 620 nm is accompanied by a growing band at 400 nm. Hiyoshi *et al.* assigned these two features to absorption from the S_1_ and T_1_ states, respectively. However, in our TEAS data, the long-wavelength wing of a band shifted to lower wavelength than the T_1_ band, is observed at time delays as short as 0.3 ps. The appearance of this feature is faster than expectations for intersystem crossing (ISC) to the triplet manifold; a plausible explanation is that, in this region, an additional S_1_ absorption band overlaps the known T_1_ band. Spectral decomposition can be accomplished with two spectral basis functions attributed to S_1_ and T_1_ absorption, as is illustrated in Fig. S15 of the ESI.† Fitting to the experimental data shows that the S_1_ basis spectrum intensity decays bi-exponentially, but one of the time components is significantly longer than our measurement window, as seen in Fig. 4. We therefore approximate this decay component by a linear function in the fits and use the gradient to estimate the associated time constant. This procedure yields time constants of *τ*_1_ = (5.7 ± 3.0) ps and *τ*_2_ = (8500 ± 900) ps in DCM. The initial excitation at 345 nm prepares N-EC (S_1_) slightly above the S_1_-state band origin, and initial relaxation occurs by vibrational energy transfer (VET) to the solvent. One possible assignment of the 5.7 ps time constant is to this VET, which might modify the ESA band shape, because of its similarity to the timescales for VET reported for other organic molecules some of which are in chlorinated solvents.^44,45^ However, we prefer an interpretation that the photoexcited S_1_ molecules can initially undergo fast intersystem crossing (ISC) to the T_1_ state in competition with VET because the T_1_ band intensity shows a similarly fast initial growth component. Thermalized N-EC (S_1_) subsequently undergoes slower ISC, and perhaps also radiative decay to S_0_, with a time constant of ∼8500 ps. Consistent with this mechanistic picture, growth of the T_1_ absorption band occurs biexponentially with an initial time constant *τ*_1_ in accord with the few-ps S_1_ decay component, and a slower contribution on timescales greater than the measurement window (*τ*_2_ > 1 ns). The corresponding measurements in ACN revealed time constants of *τ*_1_ = (9.0 ± 2.2) ps and *τ*_2_ ≫ 1 ns as seen in Fig. 4. We assign a minimum value of *τ*_2_= 10 ns in ACN for fitting purposes because the S_1_ decay is too slow for us to measure.

TEAS measurements of 7 mM 9-PC in DCM show similar features to those of N-EC, as can be seen in Fig. 3(c). A broad ESA band centred at 620 nm decays at the same rate as a second band centred near 400 nm grows. This latter feature overlaps a further ESA band appearing at wavelengths around 390 nm. By comparison with the N-EC spectra, we assign the 620 nm and 390 nm bands to ESA from 9-PC (S_1_) and the band at 400 nm to absorption from 9-PC (T_1_). T_1_ ESA growth was observed to extend over times longer than the measurement window, as seen in Fig. 4. A single time constant corresponding to ISC was estimated to have a value of *τ*_1_ = (6900 ± 200) ps using a linear fit to the decaying intensity of the main S_1_ ESA band as an approximation to the early stages of the slow exponential decay. Energetically favourable ISC from S_1_ to the T_3_ state followed by rapid internal conversion to T_1_ is suggested to occur in this system because of the CT character involving the phenyl ring in the 9-PC (T_3_) state (ESI,† Section S3 and Fig. S9), thus satisfying El Sayed's Rule.^46,47^ In contrast to observations for N-EC, fast S_1_ decay dynamics on a few ps timescale are less pronounced in 9-PC, suggesting there is not a rapid ISC pathway open to the initially excited 9-PC (S_1_) molecules in our measurements. This could simply be because 345 nm photoexcitation is closer to the band origin of 9-PC than for N-EC (see Fig. 2(a)).

Fig. 3(a) shows that three features appear in the spectral window for TVAS measurements of 7 mM N-EC in DCM. Two negative-going GSB features are centred at 1454 cm^−1^ and 1598 cm^−1^, coinciding with N-EC bands observed by FTIR in a DCM solution, and a strong positive-going band at 1479 cm^−1^ is attributed to N-EC (S_1_) ESA. The GSB features show no recovery, signifying that the S_0_ ground-state of the molecule is not repopulated on the 1.3 ns timescale of our measurements, and further supporting the population of a long-lived triplet state of N-EC with high quantum yield. However, no bands that can be attributed to T_1_ ESA are observed in the chosen mid-IR spectral window. In DCM solution, the absorption band at 1479 cm^−1^ decays biexponentially and its changing intensity can be modelled successfully with the time constants of *τ*_1_ = 5.7 ps and *τ*_2_ = 8500 ps derived from TEAS measurements as shown in the ESI† (Fig. S20).

### 3.3. Transient absorption spectroscopy of N-EC with Ph_2_I^+^PF_6_^−^ and 9-PC with Ph_2_I^+^PF_6_^−^ solutions in DCM

**Fig. 5:**
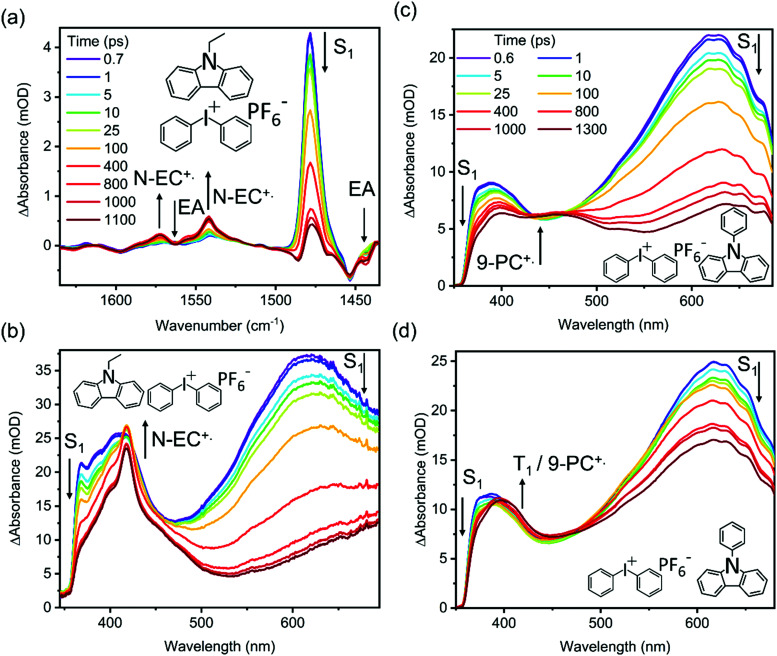
Transient absorption spectra obtained following 345 nm photoexcitation of N-EC or 9-PC in the presence of Ph_2_I^+^PF_6_^−^. Panels (a) and (b) show TVAS and TEAS data for a 7 mM N-EC and 84 mM Ph_2_I^+^PF_6_^−^ solution in DCM, respectively. Panels (c) and (d) compare TEAS data for DCM solutions of 7 mM 9-PC with 84 mM and 7 mM Ph_2_I^+^PF_6_^−^, respectively. The inset colour key identifying different delay times in (a) also applies to panel (b), and the colour key in (c) also applies to panel (d). Arrows show the directions of change of transient features.

To explore the photoinduced ET reactions of the carbazoles, TEAS spectra were obtained for 7 mM N-EC solutions in DCM or ACN with five different concentrations of Ph_2_I^+^PF_6_^−^ in the range 7–84 mM, and for a 7 mM 9-PC solution in DCM with six concentrations of Ph_2_I^+^PF_6_^−^ in the same range. Examples are shown in Fig. 5, with further data reported in the ESI† (Section S7–S9). A TVAS measurement obtained for a 7 mM N-EC and 84 mM Ph_2_I^+^PF_6_^−^ solution in DCM is also shown in Fig. 5(a). On the introduction of Ph_2_I^+^PF_6_^−^ to the N-EC solutions, the TEAS spectra change significantly from those described in Section 3.2 and illustrated in Fig. 3. The two ESA features centred near 400 nm and 620 nm are still observed, but a sharp feature centred at about 418 nm now develops. This new feature closely resembles the band seen in steady state oxidation studies of N-EC with FeCl_3_ (see ESI,† Section S6 and Fig. S18), indicating assignment to N-EC˙^+^ resulting from intermolecular ET. This assignment is supported by TDDFT calculations of the absorption spectrum of N-EC˙^+^, as shown in ESI† (Section S6 and Fig. S18). The rate of growth of this radical absorption band matches the decay of the N-EC S_1_ ESA bands, showing that under the current experimental conditions the radical forms directly from the S_1_ state of N-EC by intermolecular ET. The T_1_ ESA signature is suppressed at higher concentrations of Ph_2_I^+^PF_6_^−^ because N-EC˙^+^ formation outcompetes ISC. However, this T_1_ signature is still evident at lower concentrations of Ph_2_I^+^PF_6_^−^ because the bimolecular ET reaction from the S_1_ state becomes slower. Regardless, the contribution to the transient absorption spectra from T_1_ ESA remains small in the solutions of N-EC and the EA.

Use of a high concentration of Ph_2_I^+^PF_6_^−^ in solutions with 9-PC generates a broad, growing absorption feature which spans wavelengths from 400–500 nm, as shown in Fig. 5(c) for an 84 mM Ph_2_I^+^PF_6_^−^ solution. However, at lower Ph_2_I^+^PF_6_^−^ concentrations in our studied range (Fig. 5(d)), the TEAS spectra are more characteristic of those obtained without EA addition. These observations confirm that triplet formation in 9-PC systems occurs in competition with ET from the S_1_ excited state, and that the intermolecular ET process becomes more significant as the EA concentration increases.

The TVAS spectra measured for a solution of 7 mM N-EC and 84 mM Ph_2_I^+^PF_6_^−^ in DCM shown in Fig. 5(a) reveal new spectral features in addition to those seen for N-EC in DCM (Fig. 3). The high concentration of Ph_2_I^+^PF_6_^−^ was chosen to ensure efficient intermolecular ET. Additional negative-going features observed at 1443 cm^−1^ and 1563 cm^−1^ are assigned to depletion of Ph_2_I^+^PF_6_^−^ based on bands observed in our steady state FTIR spectra. These depletion features deepen with increasing time delay because of the removal of Ph_2_I^+^ during the ET reaction. ET to Ph_2_I^+^ is dissociative,^48,49^ hence the ET reaction with N-EC (S_1_) is likely to be irreversible, and our measurements confirm no back ET to reform N-EC (S_0_) as the assigned N-EC GSB bands show no recovery on a 1.3 ns timescale. The ESA feature at 1478 cm^−1^ corresponding to N-EC(S_1_) now decays to near-baseline in the time window of the measurements because of the ET reaction. Two additional absorption features that grow at 1542 cm^−1^ and 1572 cm^−1^ are attributed to N-EC˙^+^ formation, with this assignment supported by DFT calculations (see ESI,† Section S6 and Fig. S17).

### 3.4. Electron-acceptor concentration dependent electron transfer kinetics

The kinetics of the bimolecular ET reaction can be established by analysing the rates of loss of N-EC (S_1_) or 9-PC (S_1_) ESA and the rates of growth of N-EC˙^+^ or 9-PC˙^+^ product absorption bands in TEAS data obtained at different concentrations of EA. These measurements were made for 7 mM solutions of N-EC in DCM and ACN, with the concentration of Ph_2_I^+^PF_6_^−^ increasing from 7 mM to 84 mM. The EA was also replaced with Me_2_Ph_2_I^+^PF_6_^−^ or *t*-butyl-Ph_2_I^+^PF_6_^−^ in concentrations from 7 mM to 28 mM in DCM. Additionally, measurements were made for a 7 mM solution of 9-PC and 7–84 mM Ph_2_I^+^PF_6_^−^ in DCM.

The TEAS data were analysed using the KOALA program,^31^ with methods that are discussed in detail in the ESI,† Section S5. Time-dependent intensities of the component spectral features derived from decomposition of the TEAS data were fitted to a kinetic model based on Smoluchowski theory.^32–34^

The Smoluchowski model applies the following expression to kinetic data for the loss of excited state N-EC (denoted by N-EC*), with the same approach also used for photoexcited 9-PC (9-PC*):^32–34^1
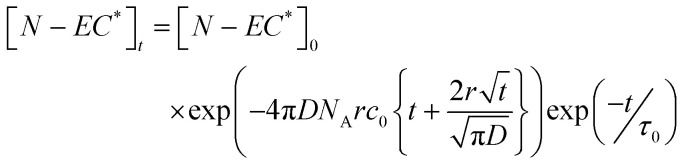


In eqn (1), [N-EC*]_0_ is the initial excited state concentration of N-EC, *c*_0_ is the concentration of the EA, and *τ*_0_ is the lifetime of N-EC (S_1_) in the absence of the EA. In this model, *k*_ET_ = 4π*DN*_A_*r* is the bimolecular rate coefficient for ET expressed in terms of the diffusion coefficient (*D*), *N*_A_ is the Avogadro constant, and *r* is the critical separation of the two molecules at which ET occurs.

A drawback with the use of the Smoluchowski fitting model outlined above in the current analysis is that it omits the fast S_1_-state decay processes that we observe at early times, with time constants of a few ps, for photoexcited N-EC in the absence of an EA (see Section 3.2.). We also recognize the possible formation of complexes between N-EC (or 9-PC) and the EA which can give rise to a fast, non-diffusive component of ET (Section 3.1). Therefore, we adopted, the modified expressions in eqn (2) and (3) for loss of reactants *R* and growth of products *P* in our data fitting to accommodate these few-ps dynamics when they were observed in data sets.2
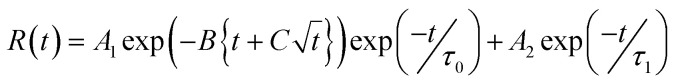
3
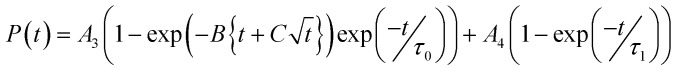


Comparison with eqn (1) shows that *B* = 4πDN_A_*rc*_0_, with dependence on the initial concentration of the EA, and 
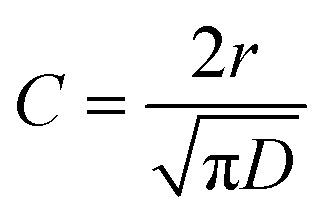
. Approximate values for *τ*_0_ were established by the TEAS studies described in Section 3.2, in which N-EC and 9-PC were studied in the absence of the EA. In the N-EC fits, we therefore fixed *τ*_0_ = 8.5 ns for DCM solutions and 10 ns in ACN, although the precise values are not important because the corresponding decay pathways are slower than the bimolecular ET reactions. For analysis of experimental data involving 9-PC, we used *τ*_0_ = 7.0 ns. The value of B was varied in all fits, but to ensure stability of the fitting, a best value of the parameter *C* was determined in fits to N-EC data sets corresponding to the highest concentrations of the EA (Ph_2_I^+^PF_6_^−^, Me_2_Ph_2_I^+^PF_6_^−^ or *t*-butyl-Ph_2_I^+^PF_6_^−^). The parameter *C* was then fixed at this value for the analysis of all other data sets corresponding to lower EA concentrations. In data sets containing 9-PC, *C* was instead selected from an average generated from lower concentrations of the EA (excluding the 84 mM and 56 mM data), because the *C* parameter was not well-determined at the higher concentrations. The error in *C* for 9-PC data was taken as one standard deviation of the determinations used for this averaging. The fast exponential decay terms with amplitudes *A*_2_ and *A*_4_ were not needed in fits to 9-PC data. For the analysis of N-EC kinetic data, a fixed value of the few-ps time constant, *τ*_1_, was taken from the fast component of decays observed in TEAS data obtained without added EA.

Fits to kinetic data sets with different initial, and excess, concentrations of the electron acceptor returned *B* values which were used in a pseudo first-order kinetic type analysis to derive the bimolecular electron transfer rate coefficient *k*_ET_ using a linear fit. The outcomes of these fits are presented in the following sub-sections for the different solutions studied. All extracted data can be found in Section S7 of the ESI.†

Uncertainties in the values of fitted parameters were taken as the standard errors from fits to eqn (2) and (3) unless otherwise stated. Uncertainties in *B* values were generated using the standard error for the highest concentration of the EA in any data set. The percentage error in *B* was then applied to the *B* values generated from data for lower concentrations of the EA. This procedure was necessary because errors in *B* values generated by fixing the *C* values in fits to eqn (2) and (3) were otherwise underestimated. Errors in derived *k*_ET_ values will also be underestimated because of the use of fixed *C* values in data fits. Therefore, a data set for N-EC and Ph_2_I^+^PF_6_^−^ in DCM was used to explore the sensitivity of *k*_ET_ to the choice of fixed *C* value. Fits were conducted with *C* chosen to take its central value of 23 ps^1/2^, or values of 40 or 15 ps^1/2^. This sensitivity analysis resulted in a range of *k*_ET_ values from which error margins were estimated and then applied to all other data sets. The analysis of kinetic data for 9-PC and Ph_2_I^+^PF_6_^−^ solutions used a modified sensitivity analysis in which the *C* values were taken to be 36 ps^1/2^ (the preferred value from the averaging of data described above), and 50 ps^1/2^ and 22 ps^1/2^. Uncertainties in *k*_ET_ values were then evaluated using the same procedure as for N-EC.

#### 3.4.1 Kinetics of electron transfer for photoexcited 9-PC and Ph_2_I^+^PF_6_^−^ solutions in DCM

**Fig. 6:**
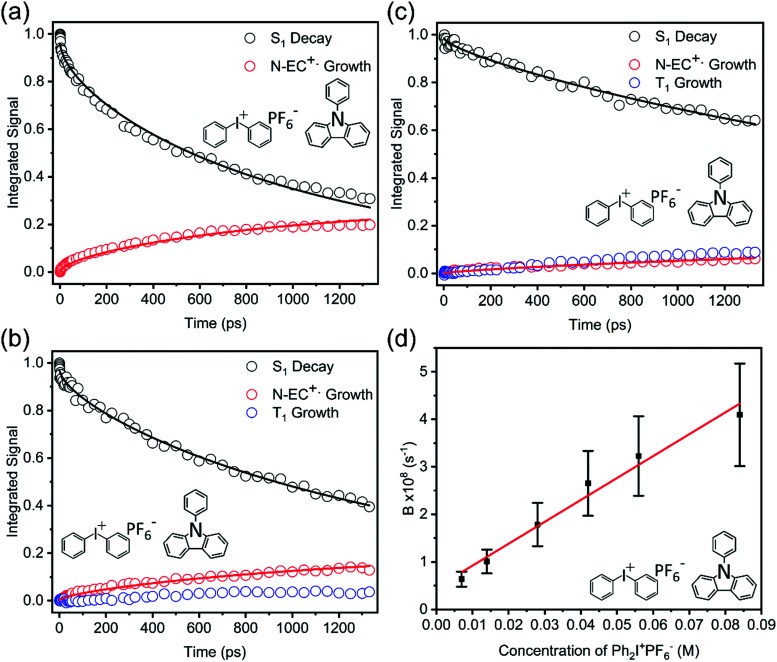
Kinetic plots obtained from TEA spectra for 345 nm photoexcitation of DCM solutions of 7 mM 9-PC and: (a) 84 mM, (b) 42 mM and (c) 14 mM Ph_2_I^+^PF_6_^−^. Coloured symbols represent 9-PC (S_1_) ESA (black), 9-PC˙^+^ formation (red), and 9-PC (T_1_) ESA (blue). Solid lines are global fits of the 9-PC (S_1_) and 9-PC˙^+^ data in each panel to the Smoluchowski model described in the main text. Panel (d) shows the pseudo-first order kinetic analysis used to determine k_ET_ from the concentration dependent B-parameter values obtained from these fits.

Analysis of 9-PC ET kinetics proved to be the most straightforward case and is therefore described first. Spectral decomposition and application of the Smoluchowski model in the form of eqn (2) and (3), without the additional fast exponential terms, to the Ph_2_I^+^PF_6_^−^ concentration-dependent kinetic analysis was sufficient to fit the data for ET reactions of 9-PC. TEAS measurements of 9-PC and Ph_2_I^+^PF_6_^−^ in DCM did not show a few-ps initial component of S_1_ population decay, which indicates that prompt ET in 9-PC complexes with Ph_2_I^+^PF_6_^−^ does not play a significant role. Fig. 5 shows the changes in the TEAS spectrum of 9-PC solutions for two different Ph_2_I^+^PF_6_^−^ concentrations, and Fig. 6 provides a kinetic analysis of band intensities from decomposed spectra. The data show that the 9-PC S_1_ state competitively decays by intermolecular ET (making 9-PC˙^+^) or ISC to the T_1_ state, with the balance of these two outcomes dictated by the concentration of the EA. At lower concentrations of the electron acceptor there is greater evidence of ISC at the expense of ET. Most of the observed ET is therefore deduced to occur from the S_1_ state in our observation time-window and under our experimental conditions of EA concentration.

The pseudo first order kinetic analysis described above obtained a bimolecular rate coefficient of *k*_ET_ = (5 ± 1) × 10^9^ M^−1^ s^−1^ in DCM. This value is smaller than, but close to the expected diffusion limited value in DCM which is estimated to be 1.6 × 10^10^ M^−1^ s^−1^ at 298 K.^50^ The ET reaction of 9-PC with Ph_2_I^+^PF_6_^−^ may therefore be sterically hindered, or have a small activation energy barrier.

#### 3.4.2 Kinetics of electron transfer for N-EC and Ph_2_I^+^PF_6_^−^ solutions in DCM

**Fig. 7:**
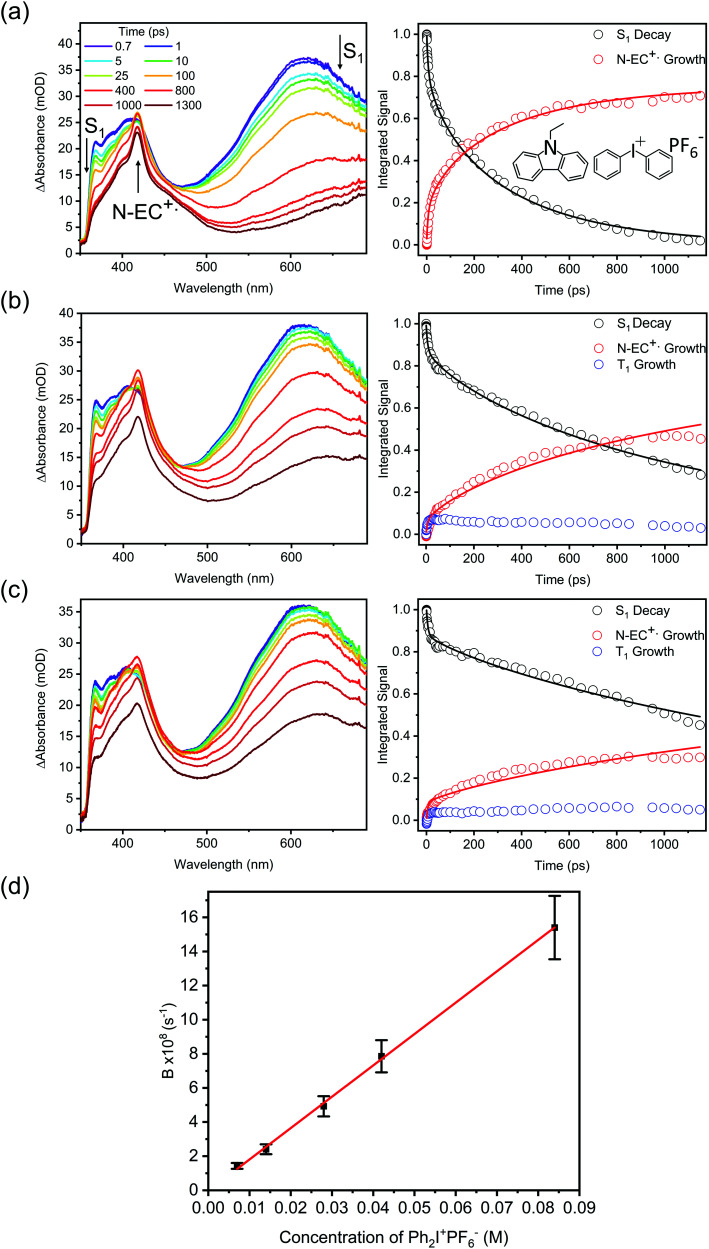
Kinetics of photoinduced ET reactions of N-EC (7 mM) and Ph_2_I^+^PF_6_^−^ (various concentrations) in DCM solutions. The left column shows TEA spectra for selected electron acceptor concentrations, and the right column shows time-dependent band intensities and kinetic fits for the decay of N-EC(S_1_) ESA (black), growth of N-EC˙^+^ absorption (red), and growth of N-EC (T_1_) ESA (blue). The concentrations of Ph_2_I^+^PF_6_^−^ were: (a) 84 mM; (b) 28 mM; (c) 14 mM. Panel (d) is a plot of the dependence of B from the Smoluchowski fitting model on concentration of the EA Ph_2_I^+^PF_6_^−^, with an unweighted linear fit. The gradient of the line of best fit is used to determine the bimolecular ET rate coefficient.

The TEAS data for N-EC and Ph_2_I^+^PF_6_^−^ in DCM, corresponding to the molecules used for polymerization studies by Sari *et al.*, proved more complicated to analyse. The time-dependence of the transient absorption spectra indicated a component of prompt (few ps) ET which is likely to occur within photoexcited complexes (as discussed in Section 3.1) as well as evidence for a fast ISC pathway in photoexcited N-EC (see Section 3.2). Our fitting to time-dependent band intensities therefore used eqn (2) and (3). The fitting results are shown in Fig. 7, and a pseudo first order kinetic analysis gives *k*_ET_ = (1.8 ± 0.5) × 10^10^ M^−1^ s^−1^. This is the largest of the ET rate coefficients obtained for the systems investigated in the current study. The *k*_ET_ values obtained for 9-PC and N-EC are compared in Sections 3.5 and 3.6. Additionally, *B* values obtained from TEAS and TVAS measurements for 7 mM N-EC and 84 mM Ph_2_I^+^PF_6_^−^ solutions in DCM were in good agreement, confirming reproducibility of the derived kinetics using different spectroscopic methods, as presented in the ESI† (Section S7).

#### 3.4.3 Kinetics of electron transfer in acetonitrile solutions

Contrary to the observations for N-EC and Ph_2_I^+^PF_6_^−^ solutions in DCM described in Section 3.4.2, solutions in ACN photoexcited with 345 nm UV light showed no clear evidence for a component of prompt ET associated with complexes between N-EC and the electron acceptor. Instead, the dominance of diffusive reactions is in accord with the analysis from Section 3.1 and the steady-state spectra of Fig. 2 showing a lower propensity for GS complexation in ACN than in DCM. However, a fast component of ISC from photoexcited N-EC (Section 3.2) needs to be incorporated into our data analysis. Therefore, eqn (2) and (3) were used to fit the time-dependent band intensities, although the additional fast exponential term was omitted in eqn (3). The value of the amplitude parameter *A*_2_ in eqn (2) was fixed relative to that of *A*_1_ using the ratio of the fast and slow decay components of the N-EC (S_1_) ESA intensity obtained from TEAS data in the absence of an electron acceptor. However, this constraint was not applied for fits to data obtained with 48 or 84 mM solutions of the EA for which the bimolecular ET reaction dominates the observed kinetics. Indeed, a simpler fit was sufficient for the data from a solution with 84 mM of EA in which eqn (2) and (3) were used without the fast exponential decay terms. Data and fits are shown in ESI,† Section S7, S8 and Fig. S21, and a pseudo first order kinetic analysis gave *k*_ET_ = (1.0 ± 0.3) × 10^10^ M^−1^ s^−1^. This outcome is comparable to the expected diffusion limited value in ACN of k_ET_ = 1.9 × 10^10^ M^−1^ s^−1^ at 298 K.^50^

#### 3.4.4 Dependence of the N-EC ET kinetics on the structure of the electron acceptor

In addition to the studies reported above with Ph_2_I^+^PF_6_^−^ as the EA, TEAS spectra were measured for 7 mM N-EC solutions in DCM with 7 mM, 14 mM and 28 mM concentrations of Me_2_Ph_2_I^+^PF_6_^−^ or *t*-butyl-Ph_2_I^+^PF_6_^−^. These studies were intended to explore the effects of greater steric bulk of the EA on the reaction kinetics. Solubility limitations prevented the study of higher concentration EA solutions. Time dependent band intensities were fitted in the same way as explained in Section 3.4.3, with all values of the amplitude factor A_2_ for the fast decay component being constrained in eqn (2). Despite the steady-state spectra plotted in Fig. 2 showing bands attributed to ground-state complexes, their concentrations are deduced to be lower than for N-EC and Ph_2_I^+^PF_6_^−^ solutions in DCM for reasons presented in Section 3.1. Consistent with the evidence from the steady-state UV-Vis spectra, no prompt component of ET corresponding to reaction within association complexes was observed following photoexcitation of the carbazole. Example TEAS data for these solutions are shown in Section S9 and Fig. S22 of the ESI.† For Me_2_Ph_2_I^+^PF_6_^−^ as an EA in reactions with photoexcited N-EC, we obtained *k*_ET_ = (1.2 ± 0.3) × 10^10^ M^−1^ s^−1^, whereas for *t*-butyl-Ph_2_I^+^PF_6_^−^, *k*_ET_ = (5.4 ± 1.5) × 10^9^ M^−1^ s^−1^. An increase in the steric bulk of the EA appears to result in a slower rate of ET as indicated by the smaller values of *k*_ET_ compared to that obtained for Ph_2_I^+^PF_6_^−^ (Section 3.4.2), perhaps because diffusion is slower for the larger EA solutes.

### 3.5 Comparison of ET rates for N-EC and 9-PC

The Gibbs energies of photoinduced electron transfer, Δ*G*_PET_, can be determined for the ET processes studied using the redox potentials of the species involved in the reaction and the calculated excited state energy of the carbazole:^6^4
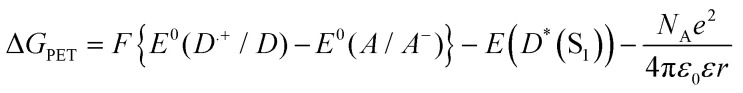


In eqn (4), *F* is the Faraday constant, *E*^0^ denotes a redox potential for the specified process, *D* and *A* are the electron donor and acceptor species respectively, and *E*(D*(S_1_)) is the S_1_-state energy of the photoexcited donor carbazole, which has undergone structural relaxation from the Franck Condon region. The final term is a work function for the charge-separated products, with ε the dielectric constant of the solvent, and *r* the distance at which ET occurs.

The values of the various terms required to evaluate Δ*G*_ET_ for each reacting couple can be found in the ESI† (Section S10). Table 1 presents the calculated Δ*G*_PET_ values alongside the experimentally measured bimolecular rate coefficients for ET. The estimated Δ*G*_PET_ values use redox potentials measured in ACN or *N*,*N*-dimethyl formamide (DMF), for which values are available in the literature; although these values will differ in DCM, they are sufficient for the purposes of our interpretation of trends in the experimental data. All the determined *k*_ET_ values for the different solutions studied are close to the diffusion limit, with the greatest deviations seen when 9-PC is the chosen carbazole and *t*-butyl-Ph_2_I^+^PF_6_^−^ is used as an EA. The ET rate coefficient for photoexcited 9-PC reaction with Ph_2_I^+^PF_6_^−^ is a factor of ∼3.5 lower than for photoexcited N-EC, despite the estimated Δ*G*_PET_ values for these two reactions being similar. The experimentally observed difference may therefore result from a greater solvation reorganization energy in the 9-PC reaction, or from steric or diffusional effects. The lower measured *k*_ET_ values for ET to *t*-butyl-Ph_2_I^+^PF_6_^−^ would also appear to be evidence of steric effects limiting close approach of the carbazole and EA, or from slower diffusion when a bulky EA is used.

**Table 1:** Thermodynamic data and the experimentally determined rate coefficients for the various electron transfer reactions studied. Redox potentials are specified for solutions in ACN with a standard calomel reference electrode (SCE). The redox potential of Ph_2_I^+^PF_6_^−^ has been corrected from the published value to correspond to that for a SCE reference electrode^56^

System	*E* ^0^(*D*˙^+^/*D*)/V (in ACN)^23,51^	*E* ^0^(*A*/*A*^−^)/*V* (in ACN)	Δ*G*_PET_ (kJ mol^−1^)	ln(*k*_ET_/M^−1^ s^−1^)
N-EC and Ph_2_I^+^PF_6_^−^ (DCM)	1.12	−1.04^48^	−568	23.6
N-EC and Ph_2_I^+^PF_6_^−^ (ACN)	1.12	−1.04^48^	−568	23.0
N-EC and Me_2_Ph_2_I^+^PF_6_^−^ (DCM)	1.12	−0.65^26^^a^	−530	23.2
N-EC and *t*-butyl-Ph_2_I^+^CF_3_SO_3_^−^ (DCM)^b^	1.12	−0.82^52^^b^	−546	22.4
N-EC and *t*-butyl-Ph_2_I^+^PF_6_^−^ (DCM)^b^	1.12	−0.2^53–55^	−483	22.4
9-PC and Ph_2_I^+^PF_6_^−^ (DCM)	1.21	−1.04^48^	−574	22.3

a The redox potential for Me_2_Ph_2_I^+^PF_6_^−^ was measured in DMF.^26^

b Two very different reported values of the redox potential for *t*-butyl-Ph_2_I^+^X^−^ in ACN have been used for comparison.^52–55,57^

### 3.6 Quantum yields for S_1_ quenching by electron transfer

The measured values for the lifetimes of the carbazole S_1_ states in the absence of an electron acceptor, *τ*(S_1_), and the bimolecular rate coefficients for reaction from the S_1_ state, *k*_ET_(S_1_), can be combined to estimate quantum yields for S_1_-state quenching by electron transfer, *Φ*_ET_(S_1_), and their dependence on the concentration of the EA, using:^58^5
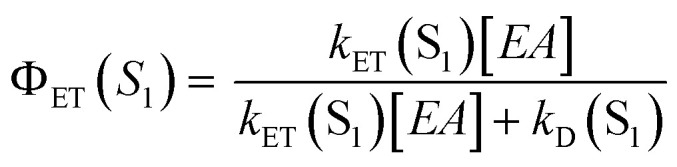


Here, *k*_D_(S_1_) = 1/*τ*(S_1_) is the unimolecular rate coefficient for decay of S_1_ population by ISC, fluorescence and non-radiative relaxation to S_0_. Using the values for *k*_ET_(S_1_) and *τ*(S_1_) determined here, Table 2 reports estimated quantum yield values for three different concentrations of the EA: 84 mM corresponds to the highest concentration and 14 mM to one of the lowest concentrations used in the current work, whereas 400 mM is the concentration used in previous polymerization studies.^1^ Further ET reaction may also occur from the carbazole T_1_ state, in particular at low concentrations of the EA. As the concentration of EA used in polymerization studies is ∼5 times larger than the highest concentration of EA used in current work it is suggested that most of the ET occurs from the carbazole S_1_ state under polymer synthesis conditions.

**Table 2:** Quantum yields *Φ*_ET_(S_1_) for photochemical electron transfer from the N-EC (S_1_) or 9-PC (S_1_) state to an electron acceptor Ph_2_I^+^PF_6_^−^ in solution

*Solution*	*k*_D_(S_1_)/(10^8^ s^−1^)	*k*_ET_/(10^10^ M^−1^ s^−1^)	*Φ*_ET_ (S_1_)		
			[EA] = 14 mM	[EA] = 84 mM	[EA] = 400 mM
N-EC and Ph_2_I^+^PF_6_^−^ in DCM	1.18 ± 0.13	1.8 ± 0.5	0.68 ± 0.23	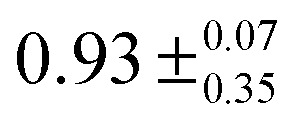	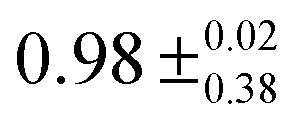
9-PC and Ph_2_I^+^PF_6_^−^ in DCM	1.45 ± 0.04	0.5 ± 0.1	0.33 ± 0.07	0.74 ± 0.19	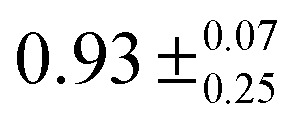

## Conclusions

The kinetics and mechanism of the initial ET step in the photoinitiated step-growth polymerization of *N*-ethylcarbazole or 9-phenylcarbazole in the presence of an electron acceptor have been studied using transient absorption spectroscopy. Decomposition of overlapping bands in transient electronic absorption spectra attributed to population of the carbazole S_1_ and T_1_ states, and formation of the carbazole radical cation, provided time-dependent concentrations of these species which were fitted to a modified Smoluchowski kinetic model. The analysis outcomes were supported by separate transient vibrational absorption spectroscopy studies. Bimolecular electron transfer was observed to occur from the S_1_ excited state of the N-EC or 9-PC under our experimental conditions. In reactions with the electron acceptor Ph_2_I^+^PF_6_^−^, the ET reaction was diffusion controlled, with deviations from this limit occurring when N-EC was replaced by 9-PC and the EA was replaced by *t*-butyl-Ph_2_I^+^PF_6_^−^. Steric effects, and perhaps also the effects of solvent reorganization energy, leading to small activation energy barriers, are suspected to be responsible for these slower reactions. Spectroscopic and kinetic evidence points to a minor role for ET reactions following photoexcitation of pre-formed, ground-state complexes between the carbazole and the EA. These complexes are most evident for solutions of N-EC and Ph_2_I^+^PF_6_^−^ in DCM, and have little influence in ACN solutions. The experimental measurements highlight the competition between bimolecular ET from the carbazole S_1_ state and intersystem crossing to the T_1_ state on timescales of a few nanoseconds. In more dilute solutions of the EA than the 7–84 mM concentrations used in the current work, ET from the T_1_ state may become important, but with a rate coefficient well below the diffusion limit.

## Data availability

Data are available at the University of Bristol data repository, data.bris, at https://doi.org/10.5523/bris.3v8qn83yngm912nqokt8sv7wt0.

## Author contributions

AJOE conceived the study, and GLT carried out the experimental measurements, data analysis, and computational calculations with assistance from RP and AJOE. GLT and AJOE wrote the paper with input from RP.

## Conflicts of interest

The authors declare no competing interests.

## Supplementary Material

CP-023-D1CP03137F-s001
