# Supplementary material for: Transient absorption spectroscopy of the electron transfer step in the photochemically activated polymerizations of N-ethylcarbazole and 9-phenylcarbazole
Source: Phys Chem Chem Phys. 2021 Aug 5;23(34):18378–92. doi: 10.1039/d1cp03137f (PMC9391922; doi:10.1039/d1cp03137f)
Supplement: CP-023-D1CP03137F-s001 [file CP-023-D1CP03137F-s001.pdf]

Electronic Supplementary Material (ESI) for Physical Chemistry Chemical Physics.
This journal is © the Owner Societies 2021

Supporting Information for:

Transient Absorption Spectroscopy of the Electron Transfer Step in the Photochemically Activated Polymerizations of N-ethylcarbazole and 9-phenylcarbazole.

Georgia L. Thornton, Ryan Phelps, Andrew J. Orr-Ewing*

School of Chemistry, University of Bristol, Cantock's Close, Bristol, BS8 1TS, U.K.

* Author for correspondence: a.orr-ewing@bristol.ac.uk

S1. Experimental Details for Steady State Measurements

All steady state UV-Vis absorption spectra were recorded using a Genesys 10S UV-Vis Spectrophotometer. Samples of N-ethylcarbazole (N-EC), 9-phenylcarbazole (9-PC), diphenyliodonium hexafluorophosphate ($Ph_2I^+PF_6^-$), bis(4-methylphenyl)iodonium hexafluorophosphate ($Me_2Ph_2I^+PF_6^-$), bis(4-tert-butylphenyl)iodonium hexafluorophosphate (t-butyl-$Ph_2I^+PF_6^-$) and solutions containing combinations of these compounds, were prepared in dichloromethane (DCM) and acetonitrile (ACN). The samples were placed separately in a Harrick cell with calcium fluoride (CaF_2) windows separated by a 250-µm Teflon spacer. The Harrick cell was positioned inside the spectrometer, a background spectrum was recorded with pure solvent, and a sample spectrum was measured across a typical wavelength range of 200-800 nm. For ground-state (GS) complex analysis, the steady state measurements were performed in a 1 cm cuvette to increase the longer-wavelength absorbance.

For all FTIR spectra recorded, a PerkinElmer Spectrum-Two FTIR spectrometer was used. The samples were prepared as above, and the method was the same as that for UV-Vis spectroscopy measurements. The FTIR spectra were record in a typical wavenumber range of 1000-4000 cm^{-1}.

For radical cation steady state spectroscopy measurements, separate samples of N-EC, 9-PC, ferric chloride ($FeCl_3$) or combinations of a carbazole and $FeCl_3$ (all 7 mM) were prepared in DCM or ACN. Spectra were taken as above in the Infrared (IR) and ultraviolet- visible (UV-Vis) regions. Radical cation bands were identified by subtraction of spectra obtained for single-solute carbazole or $FeCl_3$ solutions from those obtained for mixed solutions of the carbazole and $FeCl_3$.

S2. Steady State UV-Vis Spectra

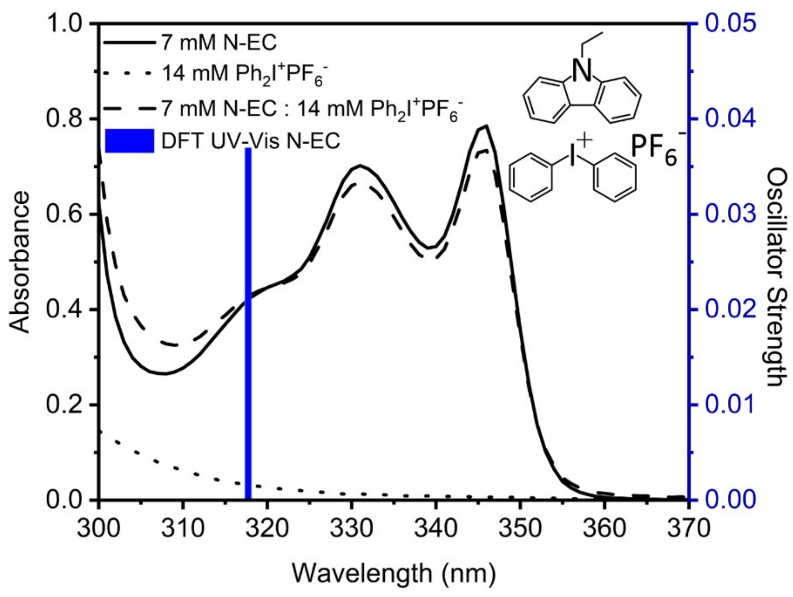

Figure S1. *UV-Vis spectra of 7 mM N-EC (solid line), 14 mM* $Ph_2I^+PF_6^-$ *(dotted line) and a 1 :2 (7 mM : 14 mM) N-EC :* $Ph_2I^+PF_6^-$ *solution (dashed line) solutions in DCM. A pump wavelength of 345 nm was subsequently chosen for transient absorption spectroscopy studies. The blue vertical line shows the computed transition wavelength and oscillator strength for the* $S_1 \leftarrow S_0$ *band of N-EC obtained using TDDFT with a B3LYP/ 6-31+G(d) level of theory.*

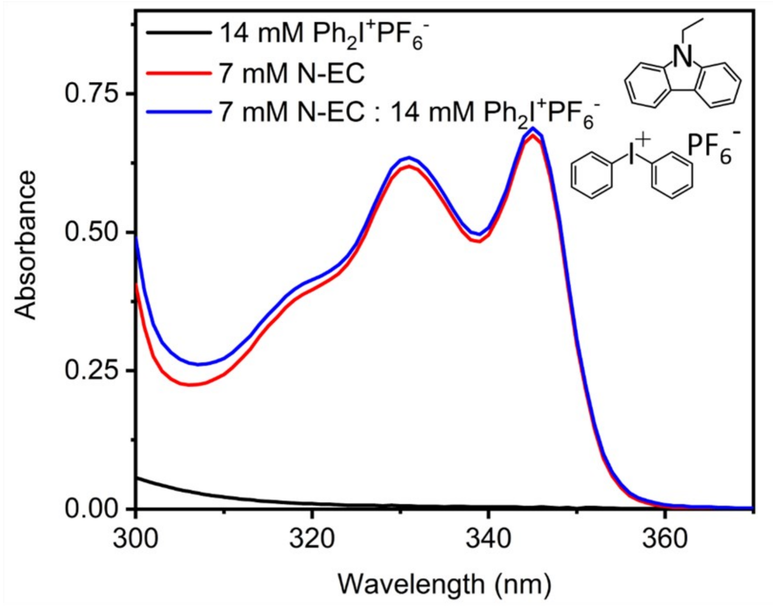

Figure S2. *UV-Vis spectra of 14 mM* $Ph_2I^+PF_6^-$ *(black), 7 mM N-EC (red) and 1: 2 (7 mM : 14 mM) N-EC :* $Ph_2I^+PF_6^-$ *(blue) solutions in ACN.*

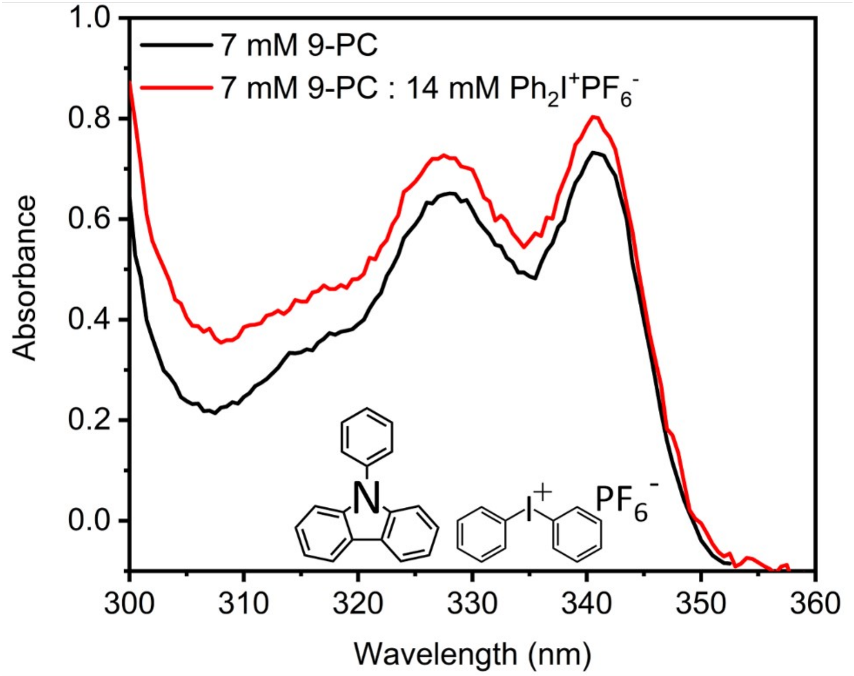

Figure S3. *UV-Vis spectra of 7 mM 9-PC (black) and 1: 2 (7 mM : 14 mM) 9-PC : $Ph_2I^+PF_6^-$ (red) solutions in DCM. The red line has been offset by -0.25 to remove effects from baseline drift.*

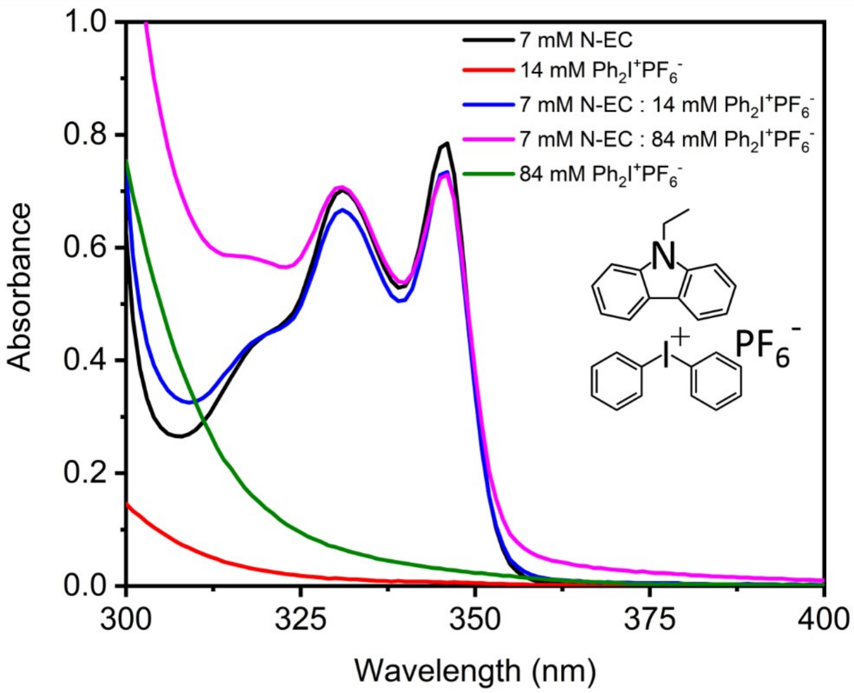

Figure S4. *Steady state UV-Vis spectra of 7 mM N-EC (black); 14 mM $Ph_2I^+PF_6^-$ (red); 7 mM N-EC and 14 mM $Ph_2I^+PF_6^-$ (blue); 7 mM N-EC and 84 mM $Ph_2I^+PF_6^-$ (pink); and 84 mM $Ph_2I^+PF_6^-$ (green) solutions in DCM.*

On addition of N-EC and $Ph_2I^+PF_6^-$ to DCM and with UV irradiation, the solution turns from pale yellow to dark blue. This colour only forms when DCM is used as the solvent of choice. When ACN is used,

the colour change is not observed. This colour change indicates a reaction between DCM and photoexcited reagents. Sari *et al.* reported that the blue colour is removed when PF_6^- ions are replaced by hydroxide anions.[1] However, the origins of this colour change remain uncertain.

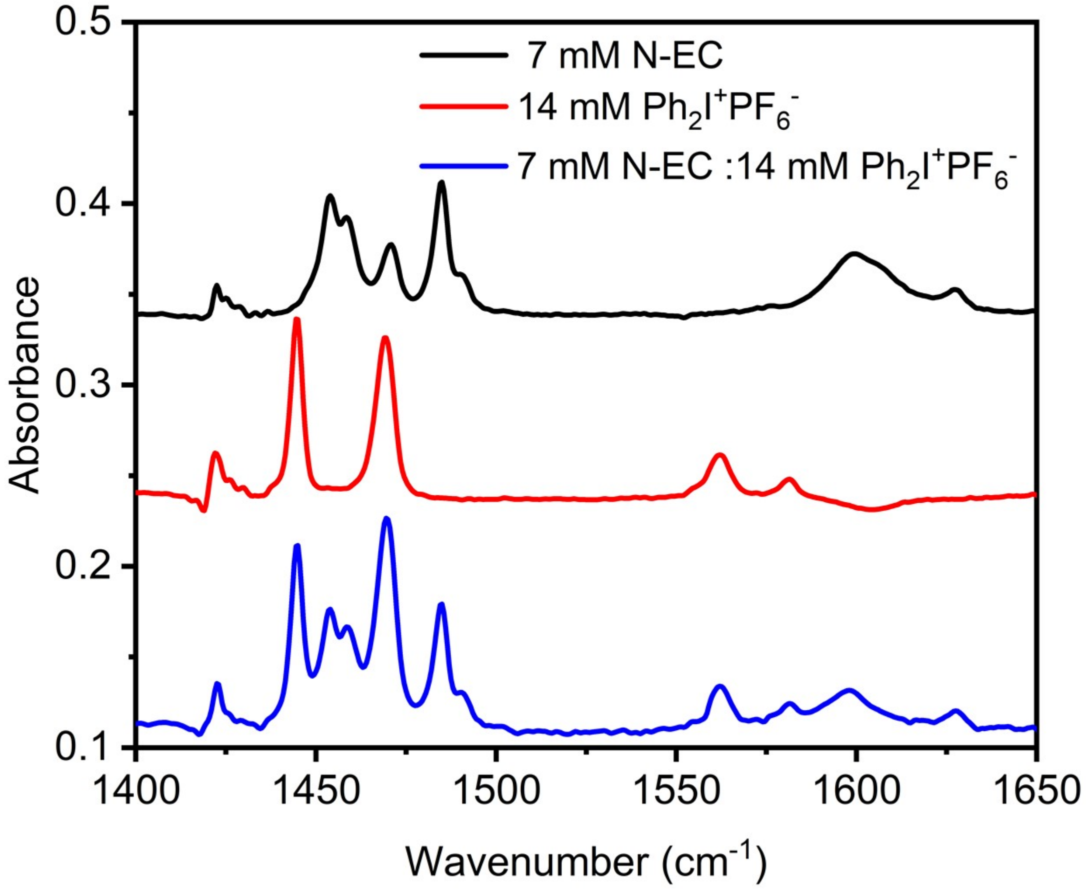

Figure S5. *FTIR spectra of 7 mM N-EC (black); 14 mM* $Ph_2I^+PF_6^-$ *(red) and 7 mM N-EC and 14 mM* $Ph_2I^+PF_6^-$ *(blue) solutions in DCM. Data have been shifted by 0.14 (black), 0.04 (red) and -0.04 (blue) vertically to see the spectral features clearly in areas of overlap, these values do not consider original baseline offset in this region.*

S3. Orbital Diagrams of Key States Involved in Transitions

N-EC

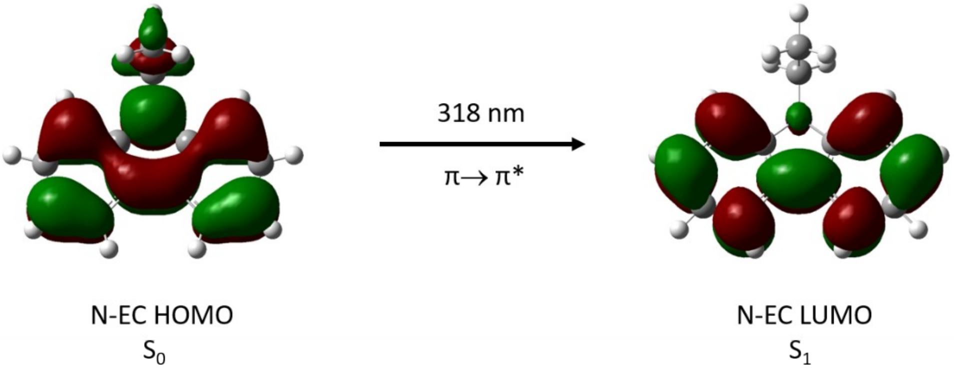

Figure S6. *Molecular orbitals involved in the Franck-Condon $S_1 \leftarrow S_0$ transition in N-EC.*

N-EC$^{+\cdot}$

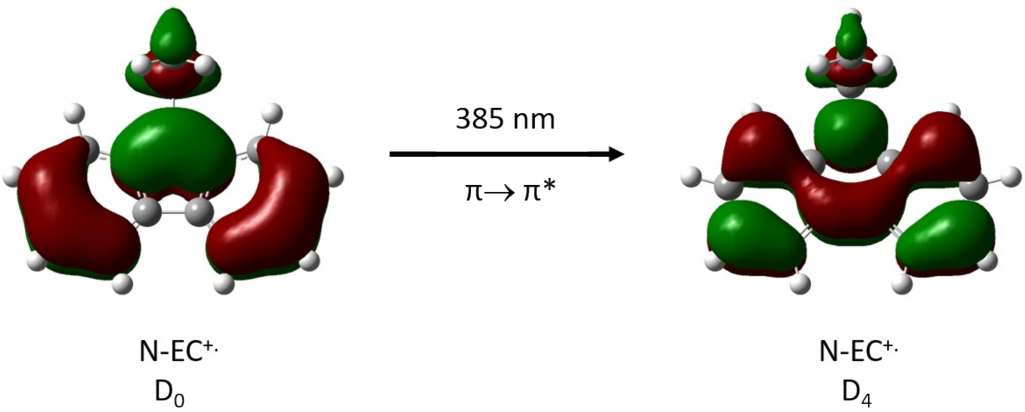

Figure S7. *Molecular orbitals involved in the Franck-Condon $D_4 \leftarrow D_0$ transition in N-EC$^{+\cdot}$. This excitation gives the characteristic absorption of the radical cation seen in TEAS spectra.*

9-PC

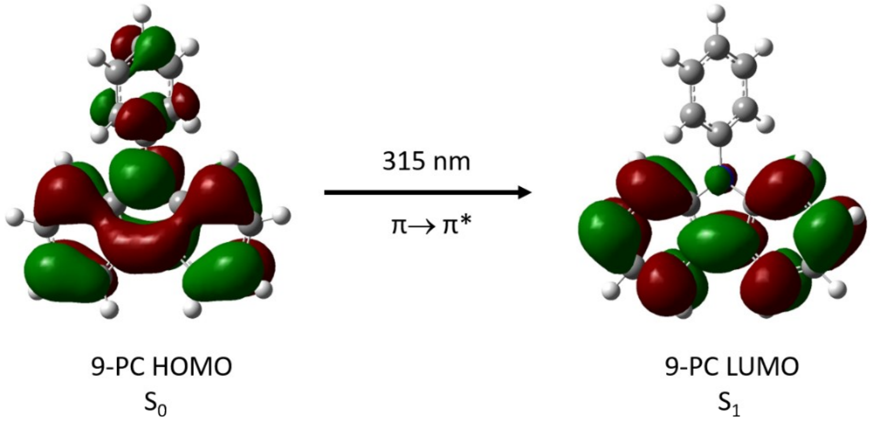

Figure S8. *Molecular orbitals involved in the Franck-Condon $S_1 \leftarrow S_0$ transition in 9-PC.*

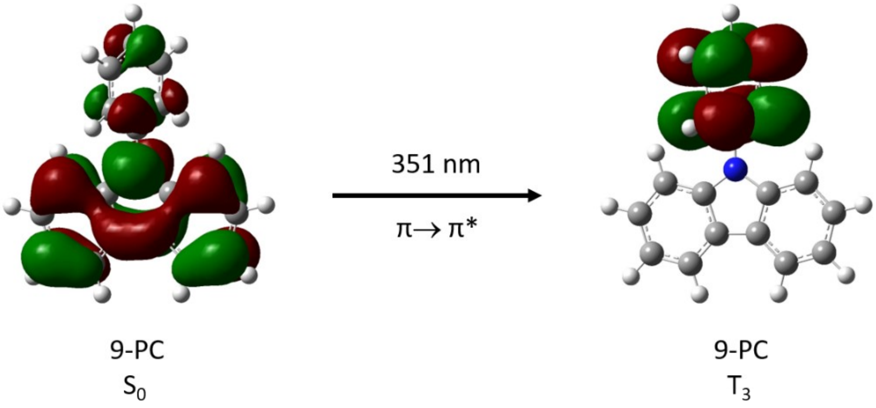

Figure S9. *Molecular orbitals involved in the $T_3 \leftarrow S_0$ transition in 9-PC.*

9-PC$^{+\cdot}$

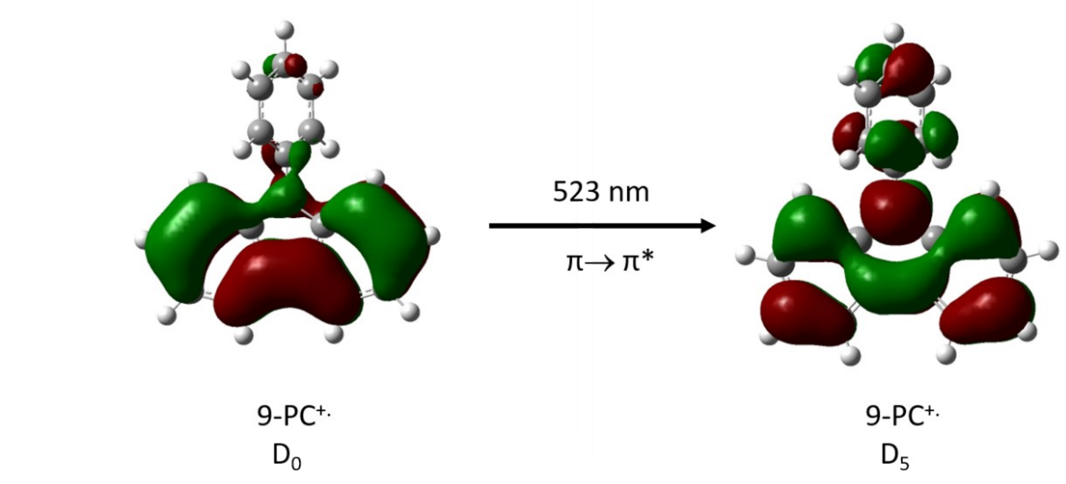

Figure S10. *Molecular orbitals involved in the Franck-Condon $D_5 \leftarrow D_0$ transition in 9-PC$^{+\cdot}$. This excitation gives one of the characteristic absorptions of the radical cation seen in TEAS spectra.*

S4. Computational Calculations for the Carbazole - EA Ground State Complex

System	Solvent	Wavelength (nm)	Oscillator Strength
N-EC and $Ph_2I^+PF_6^-$	No Solvent	324	0.026
N-EC and $Ph_2I^+PF_6^-$	ACN	321	0.040
N-EC and t-butyl$Ph_2I^+PF_6^-$	DCM	322	0.039

Table S1. *Summary of the charge transfer bands for the ground-state complexes between N-ethyl carbazole and an EA in different solvent environments.*

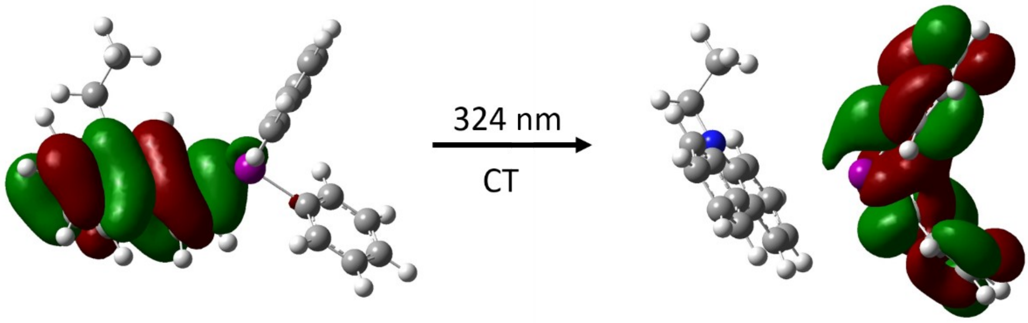

Figure S11: *Molecular orbitals involved in the Franck-Condon charge transfer transition between N-EC and* $Ph_2I^+PF_6^-$ *with calculations performed for gas-phase species. A B3LYP functional was used with a 6-31+G(d) basis set for carbon, hydrogen, and nitrogen atoms while iodine atoms were treated with a LANL2DZ basis set.*

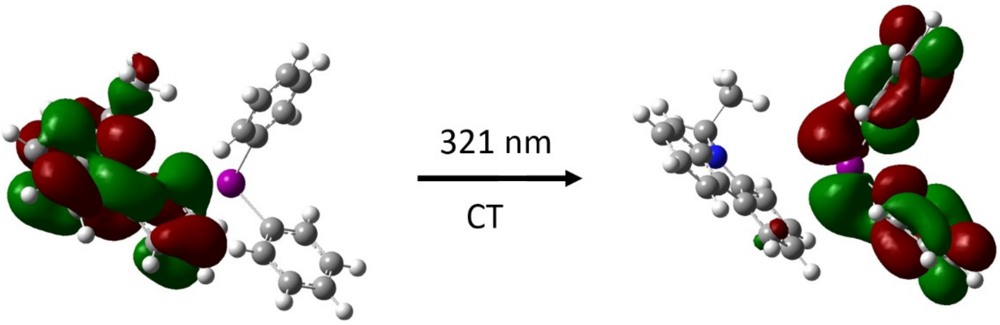

Figure S12: *Molecular orbitals involved in the Franck-Condon charge transfer transition between N-EC and* $Ph_2I^+PF_6^-$ *obtained from calculations using an IEFPCM acetonitrile solvent model. A B3LYP functional was used with a 6-31+G(d) basis set for carbon, hydrogen, and nitrogen atoms while iodine atoms were treated with a LANL2DZ basis set.*

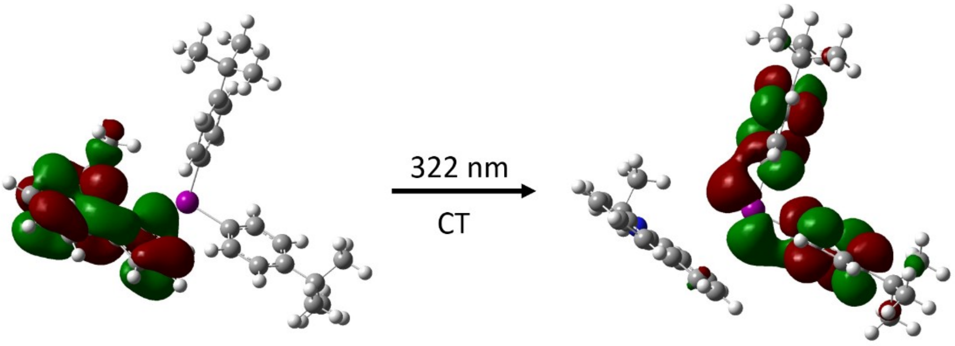

Figure S13: *Molecular orbitals involved in the Franck-Condon charge transfer transition between N-EC and t-butyl-*$Ph_2I^+PF_6^-$ *obtained from calculations using an IEFPCM dichloromethane solvent model. A B3LYP functional was used with a 6-31+G(d) basis set for carbon, hydrogen, and nitrogen atoms while iodine atoms were treated with a LANL2DZ basis set.*

S5. Analysis of Transient Absorption Spectra

Initial processing of both transient electronic absorption spectroscopy (TEAS) and transient vibrational absorption spectroscopy (TVAS) data used the KOALA program.[2] Flat-shift baseline corrections were applied to TEAS data, and quadratic baseline corrections to TVAS data. The TEAS spectra obtained at short time delays were further corrected to remove the effects of chirp in the white-light continuum probe pulse. A pre time-zero (negative time) spectrum was subtracted from all transient spectra.

TVAS data were analysed by fitting Gaussian peaks of adjustable centre and width to transient absorption and GS bleach features, and the resulting data sets of time-dependent integrated band intensities were exported as kinetic traces, as seen in figure S14.

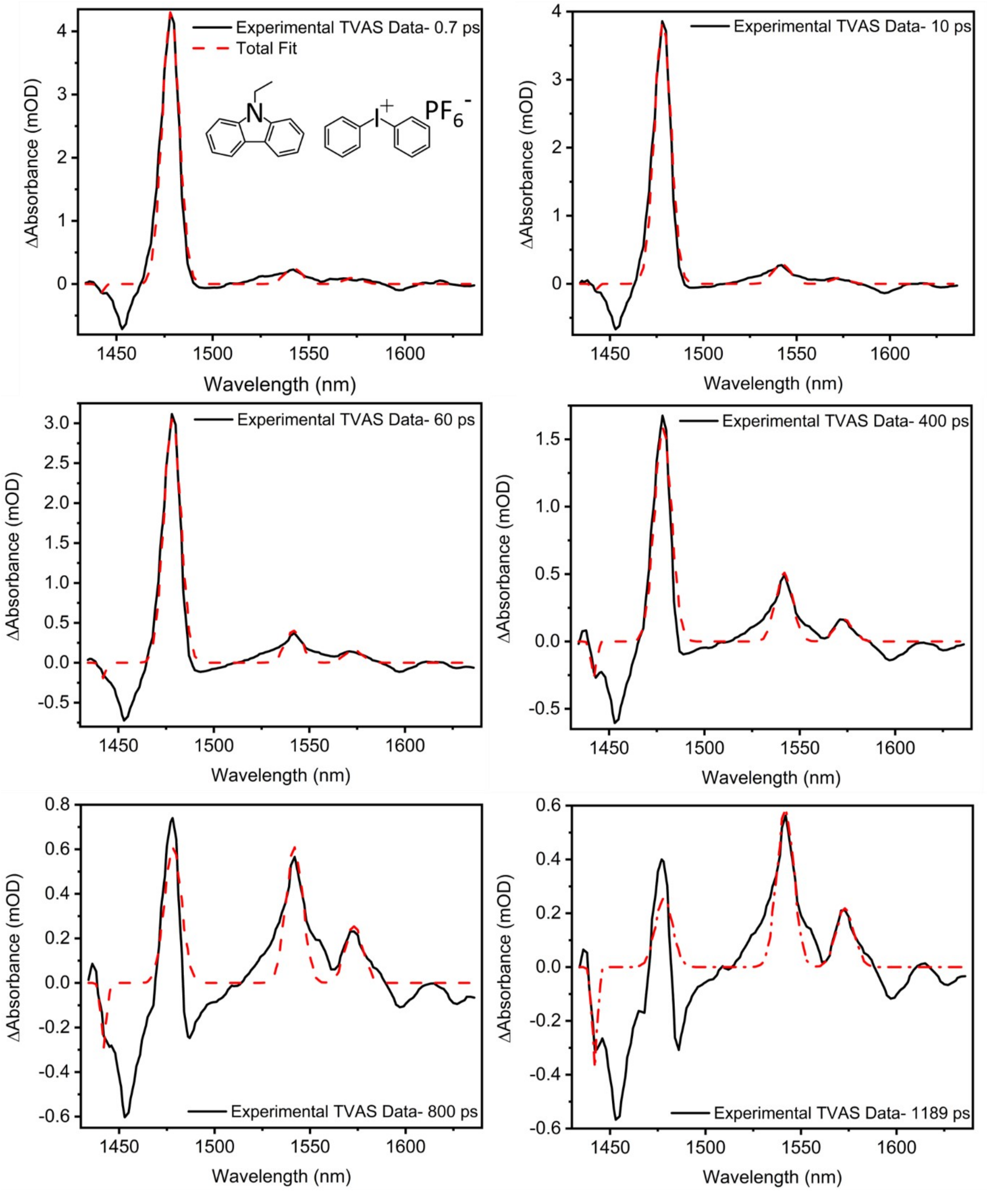

Figure S14. *Example decomposition of* TVAS *spectra obtained at time delays of 0.7 – 1189 ps for a solution of N-EC (7 mM) and* $Ph_2I^+PF_6^-$ *(84 mM) in DCM. The decomposition was carried out using Koala software.*[2] *The various panels show the experimental spectrum (black), and the overall fit (red).*

Broad and potentially overlapping bands in TEAS data were analysed using a basis spectrum decomposition method. Each system was analysed in a different way, as explained in the following sub-sections.

S5.1. N-EC / 9-PC in DCM or ACN

The analysis of all TEAS spectra relied on deriving reliable basis spectra representative of ESA from the S_1 and the T_1 states of the carbazole. These were obtained using the procedure summarized below, for each carbazole and for the two solvents (DCM and ACN) by analysis of TEAS data obtained in the absence of an electron acceptor.

The analysis windows for all spectra decompositions in the absence of EA were set to cover wavelengths typically between 380 and 660 nm. This meant that the applied basis spectra were only fitted to data within the selected analysis window. Hence spectral data outside this window did not contribute to the kinetic analysis. For the spectral decomposition, a first basis spectrum was chosen to be an experimentally measured early time TEAS spectrum, generally corresponding to a time delay of 0.3 ps. This spectrum was selected to define the carbazole S_1-state ESA signature in the absence of other species. The spectrum acquired at the largest experimental time delay was then used to prepare a basis spectrum corresponding to T_1 absorption. This late-time spectrum contained ESA contributions from both the S_1 and T_1 states because ISC is incomplete on timescales of up to 1.3 ns available in our measurements. The S_1 contribution was therefore removed using the following procedure to generate a T_1 ESA basis spectrum. First, an integration window was placed over the wavelength region from 600-650 nm where the absorption was purely from the S_1 state. The integrated signal within this window was noted either at the latest time delay, or at 550 ps (after all early time dynamics are complete) to quantify the remaining signal from the decaying S_1 state population. The early time basis spectrum (representing pure S_1 ESA) was then scaled by the fraction of the remaining S_1 state ESA in this integration window at the chosen long-time interval and subtracted from the later time spectrum to isolate the T_1 ESA component. The T_1 spectrum that is produced from this method closely resembles the T_1 spectrum reported for N-EC by Hiyoshi *et al.*[3] The resulting T_1 and S_1 basis spectra were used in the spectral decomposition performed in KOALA,[2] as illustrated in figure S15. The S_1 and T_1 ESA spectra were retained for use in the decomposition of other TEAS spectra (obtained with added electron acceptor) measured with the same carbazole and solvent. The spectral decomposition gave integrated band intensities for each time delay in TEAS measurements, which were fitted to the kinetic models described in the main text.

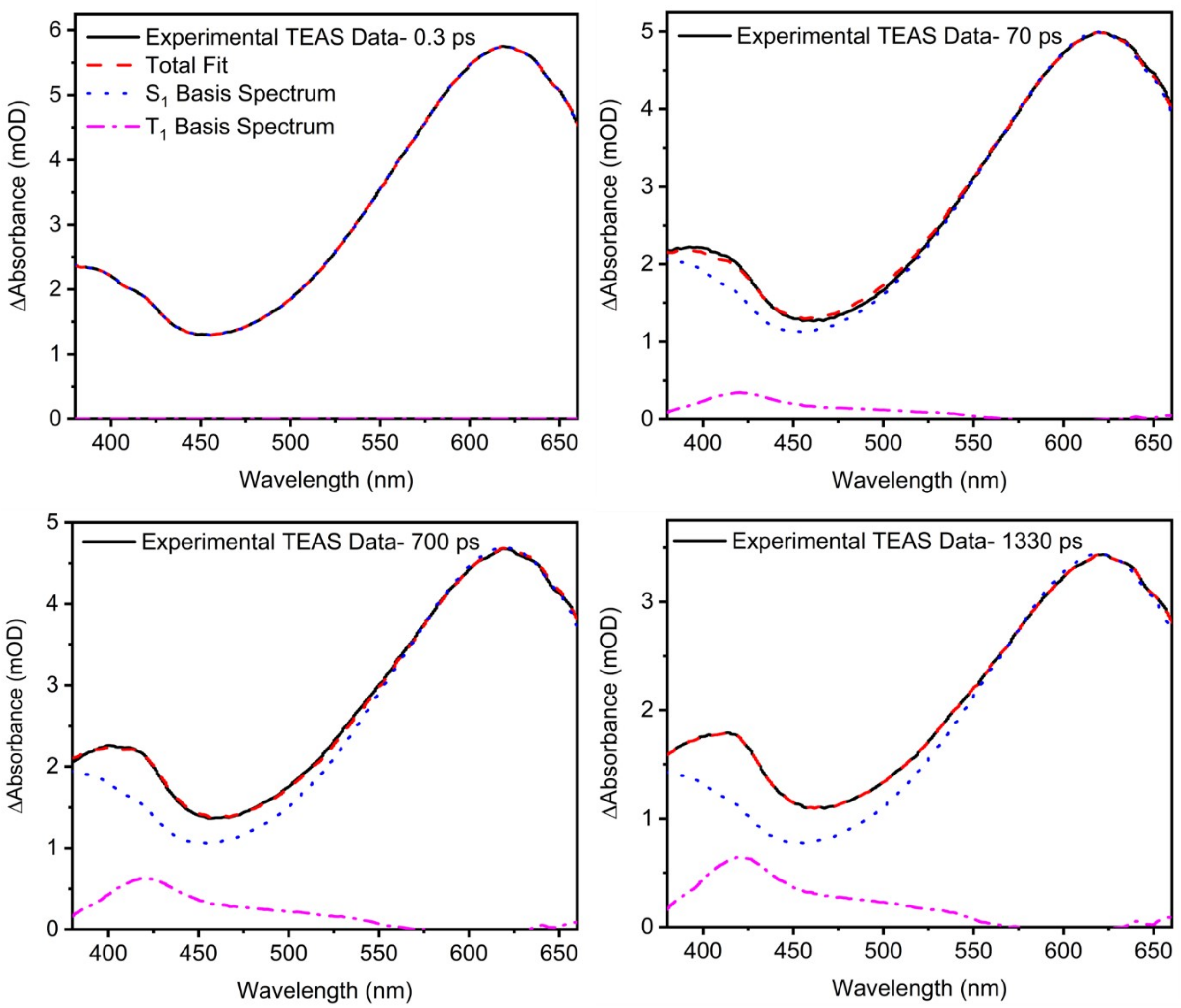

***Figure S15** Example decomposition of TEAS data obtained at several time delays from 0.3 – 1330 ps for a solution of N-EC (7 mM) in DCM. The decomposition was carried out using Koala software.[2] The panels display the experimental spectrum (black), the total fit (red), and the basis spectra created to represent the N-EC(S_1) ESA (blue) and the N-EC(T_1) ESA (pink).*

S5.2. N-EC and $Ph_2I^+PF_6^-$ in DCM

The analysis of all TEAS spectra collected upon the addition of an electron acceptor relied on deriving reliable basis spectra representative of ESA from the S_1 and T_1 states of the carbazole as well as the ground-state absorption of the radical cation. These basis spectra were obtained using the procedure summarized above for the S_1 ESA bands, and below for the radical cation absorption. T_1 ESA bands were represented by the basis spectra prepared using the procedure described in S5.1.

The basis spectrum for N-EC$^{+\cdot}$ GS absorption in DCM was obtained using the TEAS spectrum acquired at the largest time delay, and with the highest concentration (84 mM) of EA on the assumptions that

under these conditions complete ET from S_1 had occurred, leaving no residual S_1 ESA, and that the electron transfer reaction outcompeted ISC so the late-time spectrum contained no T_1 ESA component. These basis functions were used in the decomposition of TEAS spectra obtained at lower EA concentrations, with the analysis window set to 380 – 500 nm. The triplet basis spectrum was applied to low concentration of EA data and omitted from analysis of spectra obtained with 84 or 42 mM of EA because ET dominates the ISC.

S5.3. N-EC and $Ph_2I^+PF_6^-$ in ACN

The same method to obtain spectral basis functions for decomposition of TEAS data for experiments in ACN was used as described in S5.2. In the preparation of the basis functions, the analysis windows were set to cover wavelengths between 380 to 660 nm. An example of the decomposition of TEAS data for N-EC and $Ph_2I^+PF_6^-$ in ACN is shown in figure S16.

S5.4. N-EC and $Me_2Ph_2I^+PF_6^-$ in DCM

TEAS data sets for N-EC and $Me_2Ph_2I^+PF_6^-$ solutions in DCM were analysed differently to the procedures described in S5.2 and S5.3 because the highest EA concentration data set available was 28 mM. As a result, ET could not be assumed to be complete at the longest measured time delays.

The following procedure was therefore adopted. The analysis windows were set to cover wavelengths between 410-500 and 380-500 nm. A basis spectrum was made from an early time spectrum in each data set and was used to describe the N-EC S_1 ESA bands. The basis spectrum used to represent N-$EC^{+\cdot}$ was the same one as made in S5.2. However, the TEAS data had its wavelength scale recalibrated to match the absorption of the N-$EC^{+\cdot}$ feature in S5.2. The absorption of the N-$EC^{+\cdot}$ feature should be identical in all spectra for N-EC in DCM however, as the spectra were taken on different days the calibration of the wavelength axis may differ slightly. The EA does not absorb at 345 nm and therefore does not contribute any signatures to the TEAS spectra. A T_1 ESA basis spectrum was obtained as described in Section S5.1 and included in the analysis of to all data sets.

S5.5. N-EC and t-butyl-$Ph_2I^+PF_6^-$ in DCM

The limited solubility of t-butyl-$Ph_2I^+PF_6^-$ in DCM meant the same method as described in S5.4 was applied to analysis of TEAS data for this system, with the analysis window set to cover wavelengths from 390-500 nm.

S5.6. 9-PC and $Ph_2I^+PF_6^-$ in DCM

Data for 9-PC and $Ph_2I^+PF_6^-$ in DCM were analysed using a modified procedure because the ET reaction was incomplete even for 84 mM EA solutions at the largest time delay, leaving a significant ESA feature

from the S_1 state of the carbazole. Using an analysis window spanning wavelengths from 400-610 nm, the following method was adopted.

A basis spectrum was made from an early time spectrum in each data set and was used to describe the 9-PC S_1 ESA bands. A basis spectrum was also generated to represent 9-PC$^{+\cdot}$ formation with the same method used to generate the T_1-state ESA basis spectrum in S5.1 but now in the presence of 84 mM of EA. This basis spectrum was then reused for all data sets with varying EA concentrations. A T_1 ESA basis spectrum was also applied to all data sets, except for those obtained with the addition of 84 mM EA.

Time-dependent band intensity data were fitted to a version of the Smoluchowski kinetic model described in the main text for all samples containing N-EC or 9-PC and an EA.[4–6] Mono-exponential fitting provided an incomplete description of the kinetic data sets, because of contributions from static and diffusional limits of electron transfer reactions. A bi-exponential fit provided a reasonable alternative, but the Smoluchowski model analysis was preferred.

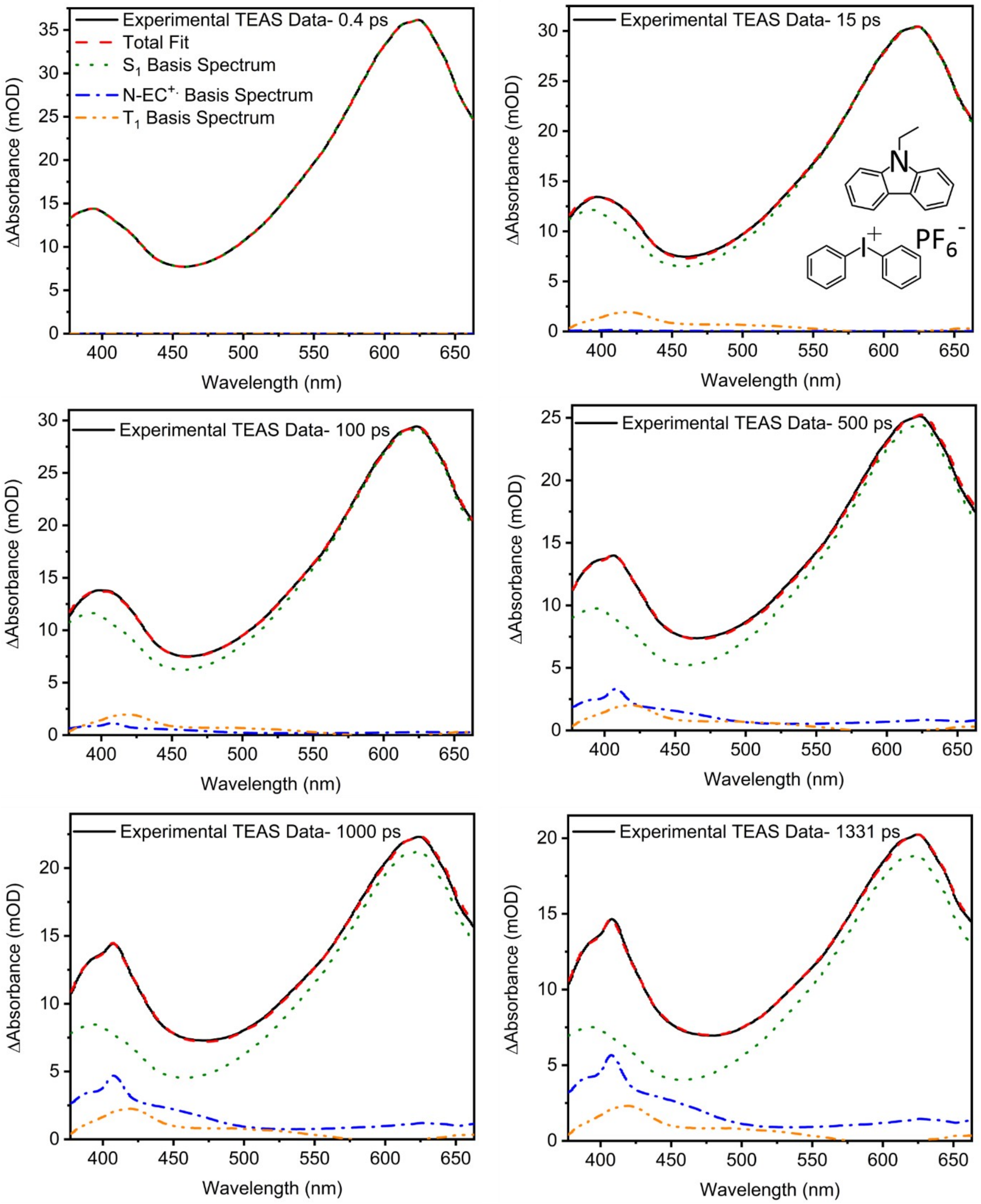

Figure S16. *Example decomposition of TEAS data obtained at several time delays from 0.4 – 1331 ps for a solution of N-EC (7 mM) and* $Ph_2I^+PF_6^-$ *(14 mM) in ACN. The decomposition was carried out using Koala software.*[2] *The panels display the experimental spectrum (black), the overall fit (red), and the basis spectra representing the N-EC(*S_1*) excited state absorption (ESA) (green), N-EC*$^{+\cdot}$ *absorption (blue), and* T_1 *ESA (orange).*

S6. Comparison of DFT Calculations and Steady State Spectra

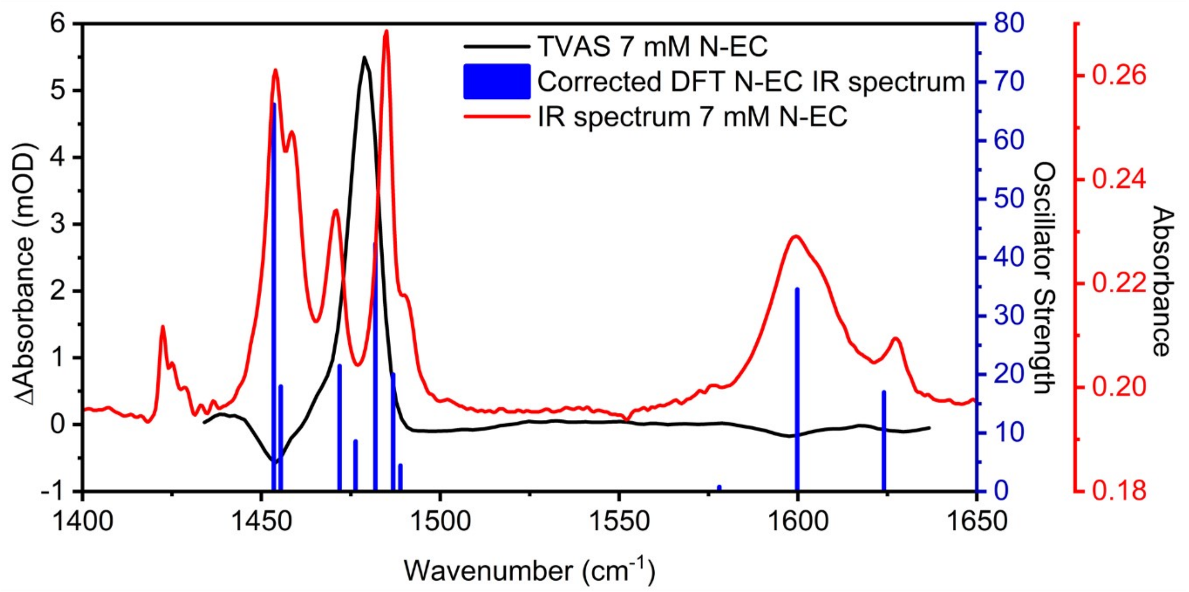

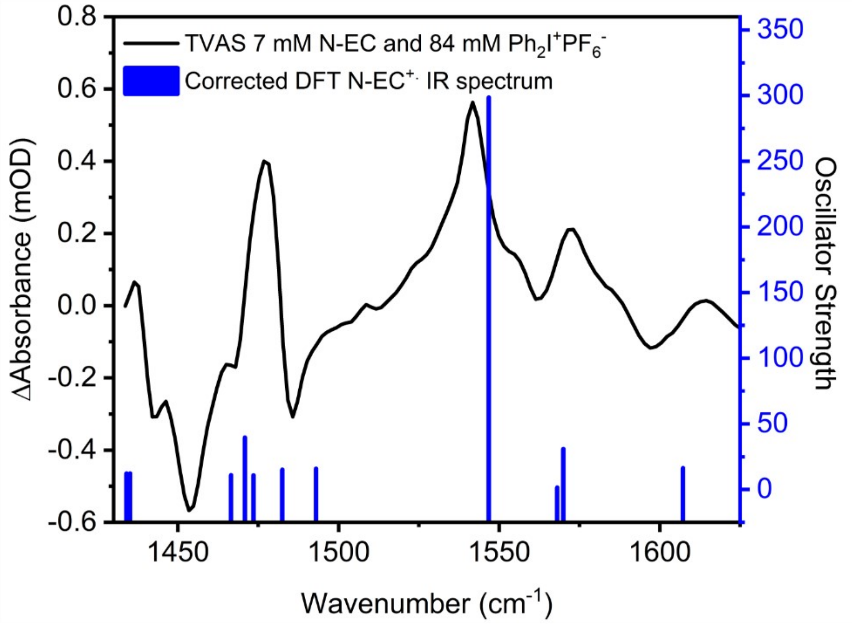

***Figure S17**. Experimental and simulated steady state IR spectra of (a) N-EC and (b) N-EC$^{+\cdot}$ and comparisons to TVAS measurements. All experimental spectra were collected in DCM, but DFT calculations were performed for an isolated gas-phase molecule. The black line in (a) is the early time (0.5 ps)* TVAS *spectrum of a 7 mM N-EC solution, and in (b) is the late time (1189 ps) TVAS spectrum for a solution of 7 mM N-EC and 84 mm* $Ph_2I^+PF_6^-$. *The blue bars depict a ground-state DFT calculated IR spectrum of (a) N-EC and (b) N-EC$^{+\cdot}$ both of which have frequencies corrected by a factor of 0.973 determined by comparison with the steady-state IR spectrum of N-EC. The red line in (a) is the FTIR spectrum of a solution of 7 mM N-EC in DCM, with absorption bands that match the ground-state bleach features seen by TVAS.*

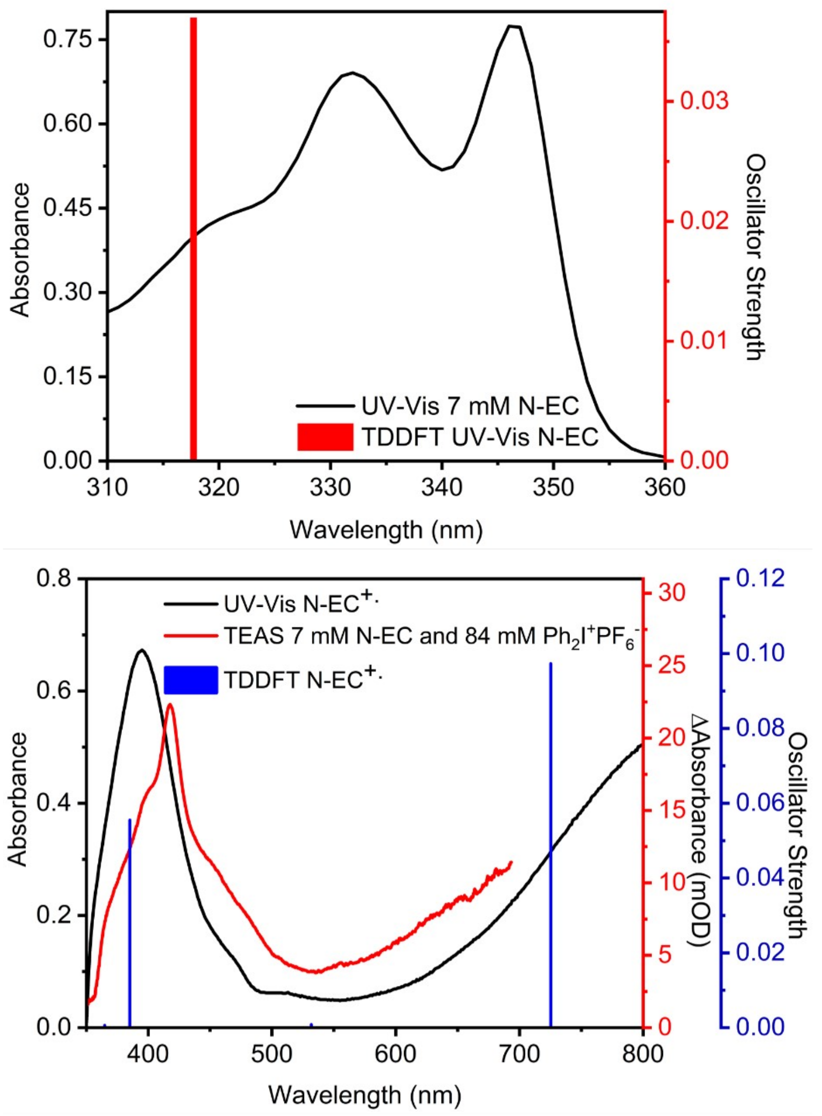

Figure S18. *Experimental and simulated UV-Vis spectra of (a) N-EC and (b) N-EC*$^{+\cdot}$. *All experimental spectra were collected in DCM, whereas TDDFT calculations were performed for a gas-phase molecule. The black line is the UV-Vis spectrum of (a) 7 mM N-EC or (b) N-EC*$^{+\cdot}$. *The red line in (b) is the late time (1372 ps) TEAS spectrum for a solution of 7 mM N-EC and 84 mM* $Ph_2I^+PF_6^-$. *The (a) red bars and (b) blue bars depict TDDFT-calculated excitations from the ground-states of (a) N-EC and (b) N-EC*$^{+\cdot}$.

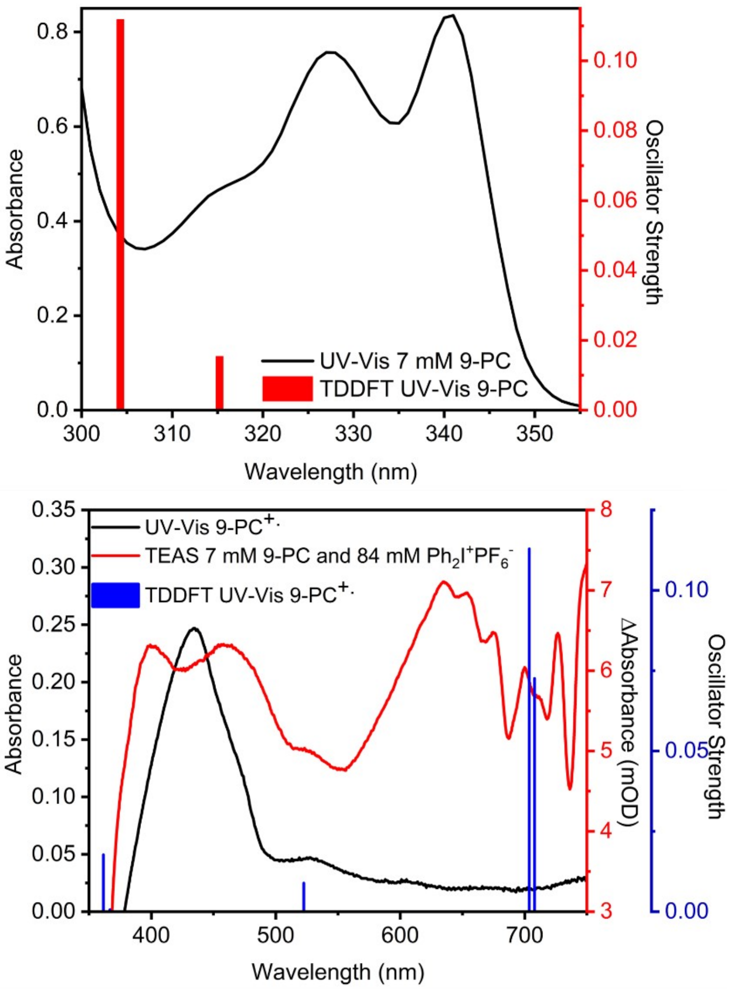

Figure S19. *Experimental and simulated UV-Vis spectra of (a) 9-PC and (b) 9-PC$^{+\cdot}$. All experimental spectra were collected in DCM, whereas TDDFT calculations were performed for a gas-phase molecule. The black line is the UV-Vis spectrum of (a) 7 mM 9-PC or (b) 9-PC$^{+\cdot}$. The red line is the late time (1331 ps) TEAS spectrum for a solution of 7 mM 9-PC and 84 mM* $Ph_2I^+PF_6^-$ *containing overlapping contributions from 9-PC (S_1) and 9-PC$^{+\cdot}$. The (a) red bars and (b) blue bars depict TDDFT-calculated UV-VIS excitations from the ground-states of (a) 9-PC and (b) 9-PC$^{+\cdot}$.*

S7. TEAS and TVAS Fitting Results

S7.1. TEAS Fitting Results

Experiment Reagents	Solvent	Species	τ_1 (ps)	τ_2 (ps)
7 mM N-EC	DCM	N-EC (S_1)	5.7 ± 3.0	8500 ± 900
7 mM N-EC	DCM	N-EC (T_1)	5.7	>> 1000
7 mM N-EC	ACN	N-EC (S_1) and N-EC (T_1)	9.0 ± 2.2	>> 1000
7 mM 9-PC	DCM	9-PC (S_1)	6900 ± 200	-
7 mM 9-PC	DCM	9-PC (T_1)	>> 1000	-

Table S2. *Summary of time-constants derived from fits to kinetic data extracted from TEAS spectra of N-EC and 9-PC in DCM and ACN solutions.*

Experiment Reagents	Solvent	B / 10^{-4} (ps^{-1})	C ($ps^{1/2}$)	k_{ET} / 10^{10} (M^{-1} s^{-1})
7 mM N-EC : 7mM $Ph_2I^+PF_6^-$	DCM	1.4 ± 0.2	23	1.8 ± 0.5
7 mM N-EC : 14 mM $Ph_2I^+PF_6^-$	DCM	2.4 ± 0.3	23	1.8 ± 0.5
7 mM N-EC : 28 mM $Ph_2I^+PF_6^-$	DCM	4.9 ± 0.6	23	1.8 ± 0.5
7 mM N-EC : 42 mM $Ph_2I^+PF_6^-$	DCM	7.9 ± 0.9	23	1.8 ± 0.5
7 mM N-EC : 84 mM $Ph_2I^+PF_6^-$	DCM	15 ± 2	23.0 ± 5.7	1.8 ± 0.5
7 mM N-EC : 7 mM $Ph_2I^+PF_6^-$	ACN	0.25 ± 0.05	64	1.0 ± 0.3
7 mM N-EC : 14 mM $Ph_2I^+PF_6^-$	ACN	1.0 ± 0.2	64	1.0 ± 0.3
7 mM N-EC : 28 mM $Ph_2I^+PF_6^-$	ACN	2.2 ± 0.4	64	1.0 ± 0.3
7 mM N-EC : 42 mM $Ph_2I^+PF_6^-$	ACN	3.6 ± 0.7	64	1.0 ± 0.3
7 mM N-EC : 84 mM $Ph_2I^+PF_6^-$	ACN	8.1 ± 1.5	64 ± 16	1.0 ± 0.3
7 mM 9-PC : 7mM $Ph_2I^+PF_6^-$	DCM	0.6 ± 0.2	36 ± 11	0.5 ± 0.1
7 mM 9-PC : 14 mM $Ph_2I^+PF_6^-$	DCM	1.0 ± 0.3	36 ± 11	0.5 ± 0.1
7 mM 9-PC : 28 mM $Ph_2I^+PF_6^-$	DCM	1.8 ± 0.5	36 ± 11	0.5 ± 0.1
7 mM 9-PC : 42 mM $Ph_2I^+PF_6^-$	DCM	2.7 ± 0.7	36 ± 11	0.5 ± 0.1
7 mM 9-PC : 56 mM $Ph_2I^+PF_6^-$	DCM	3.2 ± 0.8	36 ± 11	0.5 ± 0.1
7 mM 9-PC : 84 mM $Ph_2I^+PF_6^-$	DCM	4 ± 1	36 ± 11	0.5 ± 0.1
7 mM N-EC : 7 mM $Me_2Ph_2I^+PF_6^-$	DCM	0.49 ± 0.07	17	1.2 ± 0.3
7 mM N-EC : 14 mM $Me_2Ph_2I^+PF_6^-$	DCM	1.2 ± 0.2	17	1.2 ± 0.3
7 mM N-EC : 28 mM $Me_2Ph_2I^+PF_6^-$	DCM	2.9 ± 0.4	17.4 ± 6.3	1.2 ± 0.3
7 mM N-EC : 7 mM t-butyl-$Ph_2I^+PF_6^-$	DCM	0.22 ± 0.07	56	0.54 ± 0.15
7 mM N-EC : 14 mM t-butyl-$Ph_2I^+PF_6^-$	DCM	0.43 ± 0.14	56	0.54 ± 0.15
7 mM N-EC : 28 mM t-butyl-$Ph_2I^+PF_6^-$	DCM	1.3 ± 0.4	56 ± 26	0.54 ± 0.15

Table S3. *Summary of fitted values of the B, C and k_{ET} parameters derived from application of the Smoluchowski model in kinetic fits to TEAS band intensities for solutions of N-EC and 9-PC with three different electron acceptors ($Ph_2I^+PF_6^-$, $Me_2Ph_2I^+PF_6^-$ and t-butyl-$Ph_2I^+PF_6^-$) and in two solvents (DCM and ACN). Information on the derivation of uncertainties for the data presented can be found in the main paper. Where an uncertainty is omitted, the parameter value was fixed in the analysis.*

S7.2. TVAS Fitting Results

For the TVAS experiment with 7 mM N-EC solutions in DCM in the absence of EA, the kinetics were modelled with the values obtained in TEAS data for the same system, as reported in table S2 and shown in figure S20. Extracted kinetic data for 7 mM N-EC with 84 mM $Ph_2I^+PF_6^-$ in DCM were fit to equations (2) and (3), as seen in section 3.4 in the main paper, without the additional fast exponential term for equation 3. This difference was introduced because the N-EC$^{+\cdot}$ absorption band was evident at the earliest time delays used to acquire TVAS data, and the kinetic fit was not improved by inclusion of a fast exponential growth term for product formation. Instead, vertical offsets were applied in fits to all kinetic traces except the S_1 population decay to account for this initial absorption by products at 0.7 ps. In the kinetic analysis of TVAS data, values of the C, τ_0 and τ_1 parameters were fixed to the values obtained from the corresponding TEAS data analysis, as shown in table S3. A summary of the extracted parameters from the TVAS spectrum is presented in table S4 and the data analysis is shown in figure S20.

**Experiment Reagents**	**Solvent**	**B / 10^{-4} (ps^{-1})**	**C ($ps^{1/2}$)**
7 mM NEC : 84 mM $Ph_2I^+PF_6^-$	DCM	11.0 ± 0.1	23

Table S4. *Summary of B and C values extracted from a kinetic fit to a TVAS spectrum of a solution of 7 mM N-EC with 84 mM* $Ph_2I^+PF_6^-$ *in DCM. The C value was obtained from TEAS data measured for the same system, as shown in table S3.*

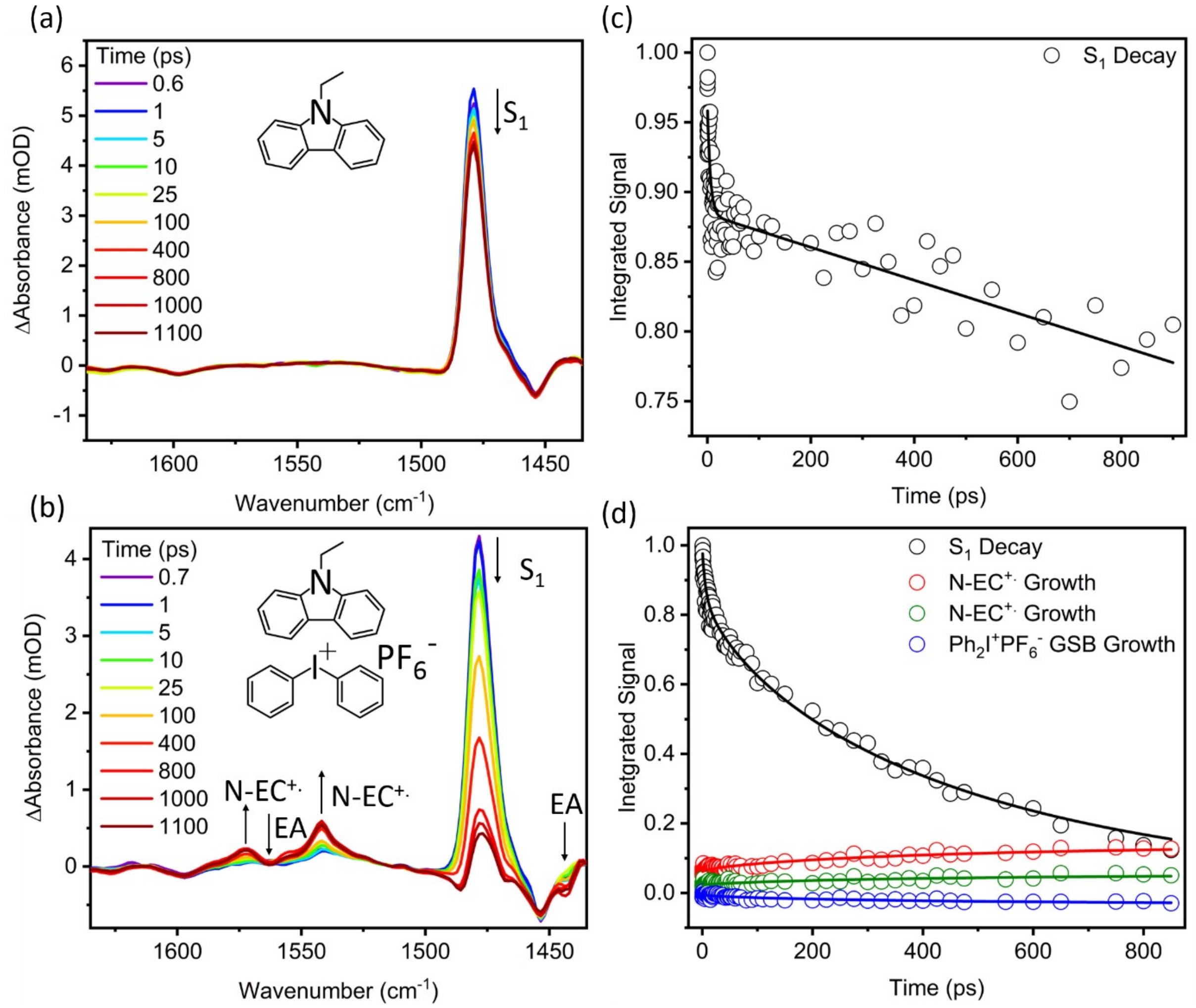

Figure S20. *TVAS spectra obtained following 345-nm photoexcitation of (a) 7 mM N-EC in DCM, or (b) 7 mM N-EC with 84 mM $Ph_2I^+PF_6^-$ in DCM. Arrows show the directions of change of transient features. Panels (c) and (d) show the corresponding kinetic traces extracted from the TVAS spectra of (a) and (b) respectively and fits to (c) a biexponential function or (d) a modified Smoluchowski fitting function. The TVAS kinetics were modelled with values from TEAS data for the same system.*

S8. Dependence of ET Kinetics on Solvent

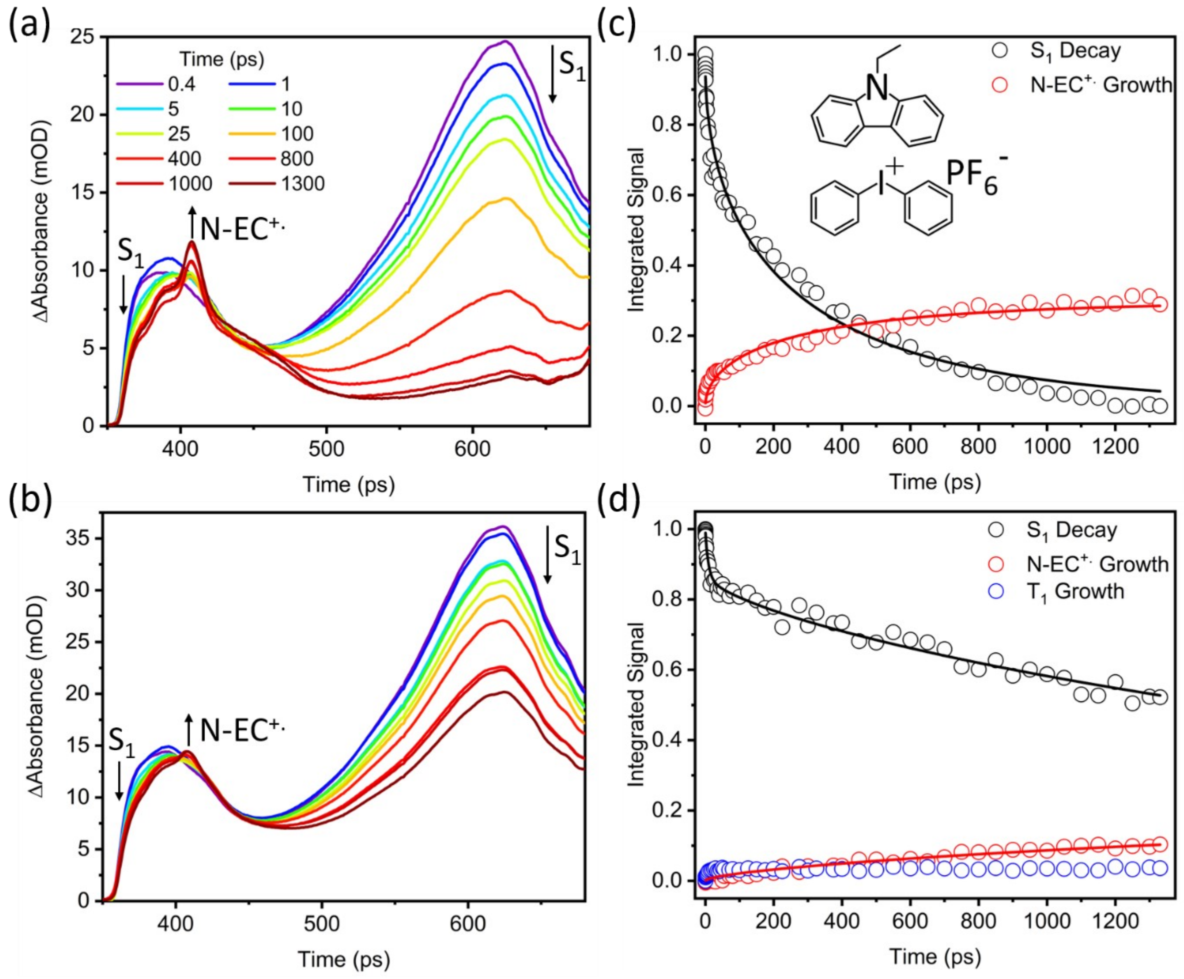

Figure S21. *TEAS spectra obtained following 345-nm photoexcitation of 7 mM N-EC and (a) 84 mM* $Ph_2I^+PF_6^-$ *or (b) 14 mM* $Ph_2I^+PF_6^-$*, in ACN. Arrows show the directions of change of transient features. Panels (c) and (d) show the corresponding kinetic traces extracted from the TEAS spectra of (a) and (b) respectively and fits to a modified Smoluchowski fitting function.*

S9. Dependence of ET kinetics on the Choice of Electron Acceptor.

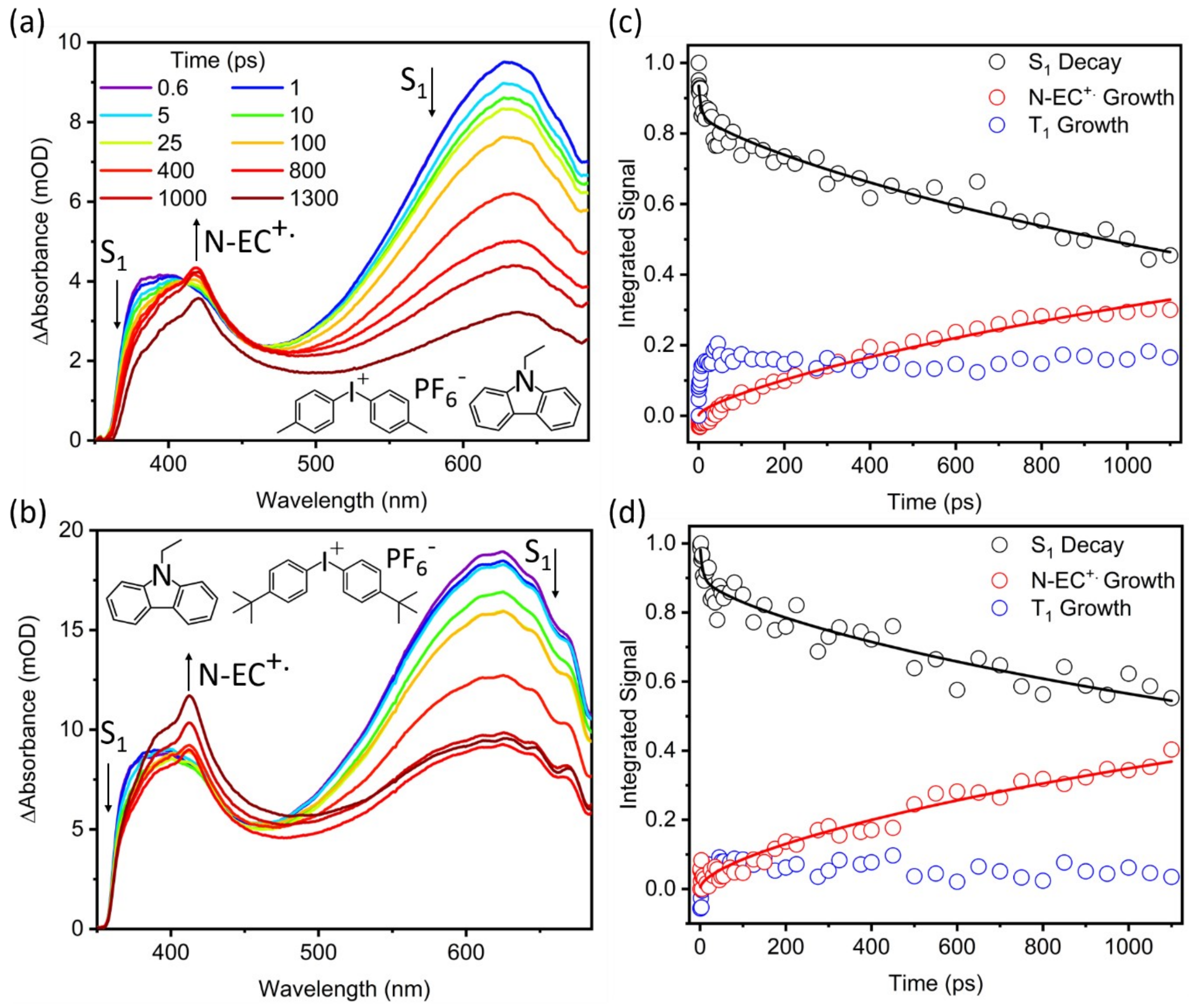

Figure S22. *TEAS spectra obtained following 345-nm photoexcitation of 7 mM N-EC and (a) 28 mM* $Me_2Ph_2I^+PF_6^-$ *or (b) 28 mM* t-butyl-$Ph_2I^+PF_6^-$*, in DCM. Arrows show the directions of change of transient features. Panels (c) and (d) show the corresponding kinetic traces extracted from the TEAS spectra of (a) and (b) respectively and fits to a modified Smoluchowski fitting function.*

S10. Gibbs Energy of Photoinduced Electron Transfer

System	$E^0(D^{\cdot+}/D)$ (V)[7,8]	$E^0(A/A^-)$ (V)	$E(D^*(S_1))$ (eV)	$E(D^*(S_1))$ (kJ mol^{-1})	ε[9] (ACN)	r (Å)	ΔG_{ET} (kJ mol^{-1})	$\ln(k_{ET})$
N-EC $Ph_2I^+PF_6^-$ in DCM	1.12	-1.04 [10]	3.68	355	36.64	10	-568	23.6
N-EC $Ph_2I^+PF_6^-$ in ACN	1.12	-1.04 [10]	3.68	355	36.64	10	-568	23.0
9-PC $Ph_2I^+PF_6^-$ in DCM	1.21	-1.04 [10]	3.66	353	36.64	10	-574	22.3
N-EC $Me_2Ph_2I^+PF_6^-$ in DCM	1.12	-0.65 [11]	3.68	355	36.64	10	-530	23.2
N-EC t-butyl-$Ph_2I^+CF_3SO_3^-$ in DCM	1.12	-0.82 [12]	3.68	355	36.64	10	-546	22.4
N-EC t-butyl-$Ph_2I^+PF_6^-$ in DCM	1.12	-0.2 [13–15]	3.68	355	36.64	10	-483	22.4

Table S5. *List of values used to determine the Gibbs energies of photoinduced electron transfer for different carbazole, electron acceptor systems. All redox potentials are specified with respect to a standard calomel electrode (SCE) in ACN apart from $Me_2Ph_2I^+PF_6^-$ which was measured in N,N-dimethylformamide (DMF).[11] Two values for the redox potential of t-butyl-$Ph_2I^+X^-$ have been reported in the literature and compared.[12–16] The redox potential of $Ph_2I^+PF_6^-$ was corrected to a SCE reference electrode.[17] S_1 donor energies were taken from time dependent TDDFT computational calculations performed for N-EC and 9-PC using a B3LYP/ 6-31+G(d) level of theory without correction for solvent effects. The S_1 state structure was optimised to determine the energy of the vibrationally cooled photoexcited donor carbazole. The distance, r, of separation between the carbazole and electron acceptor was set to be 10 Å. The work function term in equation 4 in the main manuscript did not contribute significantly to the determination of G_{ET} so a more precise r value was not needed.*

References

1 E. Sari, G. Yilmaz, S. Koyuncu and Y. Yagci, Photoinduced Step-Growth Polymerization of N-Ethylcarbazole, *J. Am. Chem. Soc.*, 2018, **140**, 12728–12731.

2 M. P. Grubb, A. J. Orr-Ewing and M. N. R. Ashfold, KOALA: A program for the processing and decomposition of transient spectra, *Rev. Sci. Instrum.*, 2014, **85**, 064104.

3 R. Hiyoshi, H. Hiura, Y. Sakamoto, M. Mizuno, M. Sakai and H. Takahashi, Time-resolved absorption and time-resolved Raman spectroscopies of the photochemistry of carbazole and N-ethylcarbazole, *J. Mol. Struct.*, 2003, **661**–**662**, 481–489.

4 M. Rini, D. Pines, B.-Z. Magnes, E. Pines and E. T. J. Nibbering, Bimodal proton transfer in acid-base reactions in water, *J. Chem. Phys.*, 2004, **121**, 9593–9610.

5 T. Kumpulainen, B. Lang, A. Rosspeintner and E. Vauthey, Ultrafast Elementary Photochemical Processes of Organic Molecules in Liquid Solution, *Chem. Rev.*, 2017, **117**, 10826–10939.

6 D. D. Eads, B. G. Dismer and G. R. Fleming, A subpicosecond, subnanosecond and steady-state study of diffusion-influenced fluorescence quenching, *J. Chem. Phys.*, 1990, **93**, 1136–1148.

7 K. Karon and M. Lapkowski, Carbazole electrochemistry: a short review, *J. Solid State Electrochem.*, 2015, **19**, 2601–2610.

8 J. F. Ambrose and R. F. Nelson, Anodic Oxidation Pathways of Carbazoles: I. Carbazole and N-Substituted Derivatives, *J. Electrochem. Soc.*, 1968, **115**, 1159–1164.

9 M. Ates and A. S. Sarac, Capacitive behavior of polycarbazole- and poly(N-vinylcarbazole)-coated carbon fiber microelectrodes in various solutions, *J. Appl. Electrochem.*, 2009, **39**, 2043–2048.

10 J. Kabatc, J. Ortyl and K. Kostrzewska, New kinetic and mechanistic aspects of photosensitization of iodonium salts in photopolymerization of acrylates, *RSC Adv.*, 2017, **7**, 41619–41629.

11 P. Chao, R. Gu, X. Ma, T. Wang and Y. Zhao, Thiophene-substituted phenothiazine-based photosensitisers for radical and cationic photopolymerization reactions under visible laser beams (405 and 455 nm), *Polym. Chem.*, 2016, **7**, 5147–5156.

12 X. Allonas, J. P. Fouassier, M. Kaji and Y. Murakami, Excited state processes in a four-component photosensitive system based on a bisimidazole derivative, *Photochem. Photobiol. Sci.*, 2003, **2**, 224–229.

13 T. N. Eren, B. Graff, J. Lalevee and D. Avci, Thioxanthone-functionalized 1,6-heptadiene as monomeric photoinitiator, *Prog. Org. Coatings*, 2019, **128**, 148–156.

14 M. Abdallah, F. Dumur, A. Hijazi, G. Rodeghiero, A. Gualandi, P. G. Cozzi and J. Lalevée, Keto-coumarin scaffold for photoinitiators for 3D printing and photocomposites, *J. Polym. Sci.*, 2020, **58**, 1115–1129.

15 A. Al Mousawi, A. Arar, M. Ibrahim-Ouali, S. Duval, F. Dumur, P. Garra, J. Toufaily, T. Hamieh, B. Graff, D. Gigmes, J.-P. Fouassier and J. Lalevée, Carbazole-based compounds as photoinitiators for free radical and cationic polymerization upon near visible light illumination, *Photochem. Photobiol. Sci.*, 2018, **17**, 578–585.

16 P. P. Romańczyk and S. S. Kurek, The Reduction Potential of Diphenyliodonium Polymerisation Photoinitiator Is Not −0.2 V vs. SCE. A Computational Study, *Electrochim. Acta*, 2017, **255**, 482–485.

17 V. V. Pavlishchuk and A. W. Addison, Conversion constants for redox potentials measured versus different reference electrodes in acetonitrile solutions at 25°C, *Inorganica Chim. Acta*, 2000, **298**, 97–102.
